# Mechanism of cooperative *N*-glycan processing by the multi-modular endoglycosidase EndoE

**DOI:** 10.1038/s41467-022-28722-w

**Published:** 2022-03-03

**Authors:** Mikel García-Alija, Jonathan J. Du, Izaskun Ordóñez, Asier Diz-Vallenilla, Alicia Moraleda-Montoya, Nazneen Sultana, Chau G. Huynh, Chao Li, Thomas Connor Donahue, Lai-Xi Wang, Beatriz Trastoy, Eric J. Sundberg, Marcelo E. Guerin

**Affiliations:** 1 Structural Glycobiology Laboratory, Biocruces Bizkaia Health Research Institute, Cruces University Hospital, 48903 Barakaldo, Bizkaia Spain; 2 Structural Glycobiology Laboratory, Center for Cooperative Research in Biosciences (CIC bioGUNE), Basque Research and Technology Alliance (BRTA), Bizkaia Technology Park, Building 801A, 48160 Derio, Spain; 3 Department of Biochemistry, Emory University School of Medicine, Atlanta, GA 30322 USA; 4 Department of Chemistry and Biochemistry, University of Maryland, College Park, MD 20742 USA; 5 Ikerbasque, Basque Foundation for Science, 48009 Bilbao, Spain

**Keywords:** Glycobiology, Enzyme mechanisms, X-ray crystallography, Bacterial structural biology

## Abstract

Bacteria produce a remarkably diverse range of glycoside hydrolases to metabolize glycans from the environment as a primary source of nutrients, and to promote the colonization and infection of a host. Here we focus on EndoE, a multi-modular glycoside hydrolase secreted by *Enterococcus faecalis*, one of the leading causes of healthcare-associated infections. We provide X-ray crystal structures of EndoE, which show an architecture composed of four domains, including GH18 and GH20 glycoside hydrolases connected by two consecutive three α-helical bundles. We determine that the GH20 domain is an exo-β-1,2-*N*-acetylglucosaminidase, whereas the GH18 domain is an endo-β-1,4-*N*-acetylglucosaminidase that exclusively processes the central core of complex-type or high-mannose-type *N*-glycans. Both glycoside hydrolase domains act in a concerted manner to process diverse *N*-glycans on glycoproteins, including therapeutic IgG antibodies. EndoE combines two enzyme domains with distinct functions and glycan specificities to play a dual role in glycan metabolism and immune evasion.

## Abstract (web summary)

EndoE is a multi-domain glycoside hydrolase of the human pathogen *Enterococcus faecalis*. Here, the authors present crystal structures of EndoE and provide biochemical insights into the molecular basis of EndoE’s substrate specificity and catalytic mechanism.

## Introduction

Glycans play a central role in bacterial physiology and pathogenicity. They are used primarily as structural constituents of bacterial cell envelopes as well as metabolic intermediates and molecules that allow energy storage^1–5^. Glycans, in the form of exopolysaccharides, are considered the fundamental components that determine the structural integrity of bacterial biofilms^6,7^. Moreover, glycan modification of proteins and lipids generates substantial structural diversity in bacteria. These structures play critical roles in molecular recognition events including the evasion of the immune response and host-pathogen interactions.

Glycans are broken down and released from carbohydrate and non-carbohydrate moieties into their constituent monosaccharides by enzymes known as glycoside hydrolases (GHs). The diversity of GHs is astounding, with well more than a hundred GH families whose members hydrolyze distinct glycan bonds^8^, as is the diversity of the organisms that produce them. The majority of GHs currently known derive from bacteria, which mainly use these enzymes to liberate and metabolize glycans from their environment as a food source and to remodel their cell envelope^2,9–12^. Some bacteria also leverage GHs to modulate host glycans as an immune evasion mechanism^13,14^.

Most GHs from bacteria comprise a single domain that is responsible for glycan hydrolysis. Many GHs do contain an extra non-GH domain known as a carbohydrate-binding module (CBM), the predicted function of which is to localize the substrate^15^, while still fewer contain additional domains that can direct the enzyme to certain glycoprotein targets^16^. Although even rarer, some GHs contain multiple GH domains with distinct glycan specificities. Such multidomain enzymes likely evolved to enhance the organism’s glycan metabolizing activities. One example of such a multidomain GH enzyme is found in *Caldicellulosiruptor bescii* and contains both GH10 and GH48 domains, which process xylan and cellulose, respectively^17^. EndoE, from *Enterococcus faecalis*, also contains two predicted GHs, each of which belongs to the endo-β-*N*-acetylglucosaminidase (ENGase) GH18 and hexosaminidase GH20 families^18^. Like the development of multidomain GHs in plants, such carbohydrate-modifying enzymes (CAZymes)^19^ in bacteria, including EndoE, are most likely the result of evolutionary gain-of-function pathways, where transduction may also have been involved, to enhance the glycan processing capabilities of the organism^20^. Whether the two GH domains with their distinct glycan specificities joined in a single polypeptide function in a concerted manner or perform their glycan hydrolysis reactions independently, but still confer an evolutionary advantage to the organism by doing so at the same time and place, remains unclear.

*E. faecalis*, an opportunistic human pathogen, especially in nosocomial environments, is known to cause a variety of human diseases including pharyngitis, skin infection, more severe infections such as toxic shock syndrome, and the non-infectious sequelae acute rheumatic fever and rheumatic heart disease, together resulting in more than 500,000 deaths worldwide each year^21–23^. Treatment of enterococcal infections is complicated by the potential development of antibiotic resistance^24^. Besides its role in nutrient acquisition, EndoE has been proposed to revert the biofilm inhibiting effect of lactoferrin^25^, and may also be involved in immune evasion by removing the glycan on human Immunoglobulin G (IgG) antibodies^18,26,27^, thereby preventing engagement of IgG antibodies with Fc gamma receptors and complement, to suppress the immune response to infection. This has been found to be the function of EndoS and EndoS2, IgG-specific GH18s from *Streptococcus pyogenes* that cleave the glycosidic bond between the first and second GlcNAc residues on glycans located at the conserved Asn297 residue on antibodies^13^.

**Fig. 1:**
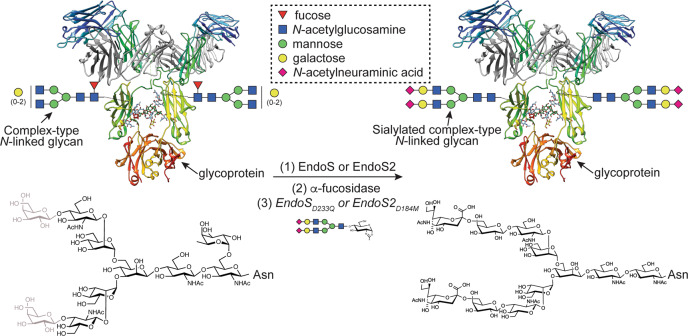
IgG glycoengineering by ENGases. ENGases can be used to remodel the glycosylation pattern of IgG antibodies. In the first step, an ENGase is used to cleave the variety of glycoforms present in the IgG antibody. In the second step, additional enzymes such as α-fucosidase can be used to remove specific glycan moieties that are not cleaved by the ENGase. In the third step, a glycan-oxazoline donor is used along with an ENGase glycosynthase mutant in order to transfer a specific glycan and obtain a homogeneously *N*-glycosylated IgG antibody.

The presumptive multidomain cooperativity of multidomain GHs, such as EndoE, may also have applications in chemoenzymatic synthesis, by which glycans on glycoproteins, such as IgG antibodies, can be remodeled. The *N*-linked glycan on the Asn297 glycosylation site typically corresponds to a biantennary complex-type (CT)^28^ but IgG antibodies, including those for clinical use, display a considerable degree of structural heterogeneity existing as a mixture of several glycoforms^29,30^. This heterogeneity has a remarkable impact on their efficacy and their effector functions. One technique to produce a custom glycoform involves expressing IgG antibodies in host expression systems with an engineered glycan biosynthetic pathway, such that the production of a major type of glycoform is favored^31,32^. However, the quality and diversity of glycoforms produced with this approach are limited due to the complexity of the *N*-linked glycan biosynthetic pathways. Chemoenzymatic glycan remodeling represents an alternative to circumvent these difficulties and generates high yields of homogeneously glycosylated IgG antibodies by using ENGases^33,34^. If two reaction products are present (*i.e*., a protein with a GlcNAc residue attached and a hydrolyzed *N*-glycan), ENGases can catalyze the reverse reaction and restore the glycosidic bond^35^. Using ENGase mutants that display glycosynthase activity and a *N*-glycan oxazoline that acts as an activated glycosyl donor substrate can further enhance the reverse reaction (Fig. 1)^36^. EndoE has been shown to facilitate the release of high-mannose type (HM-type) *N*-glycans from RNAse B and CT *N*-glycans from IgG and lactoferrin^18,25^ and it exhibits remarkable deglycosylation activity on recombinant trastuzumab harboring Man_5_ HM-type *N*-glycans produced on engineered yeast, showing its potential usefulness in the glycosylation remodeling of monoclonal IgG antibodies^26^. Enzymes with two or more GH domains fused together by an amino acid linker, such as in EndoE, may expand the combinations of glycoside hydrolases and/or glycosynthases that could be used in one-pot reactions to produce glycoproteins with custom glycoforms.

In this work, we provide high-resolution crystal structures of (i) the N-terminal GH18 domain of EndoE, both in its unliganded form and in complex with the Man_5_ product, (ii) the two three α-helical bundle linker domains, and (iii) the C-terminal GH20 domain in its unliganded form. In combination with site-directed mutagenesis, *N*-glycoprotein chemistry, enzyme activity, and kinetics, small-angle X-ray scattering (SAXS), and computational modeling, we unveil the molecular basis of distinct substrate specificity in each of the two EndoE GH domains, as well as define the mechanism by which these two GH domains act in concert to process *N*-glycans that neither individual GH domain could hydrolyze independently.

## Results

### Structure of the GH18 domain of EndoE

**Fig. 2:**
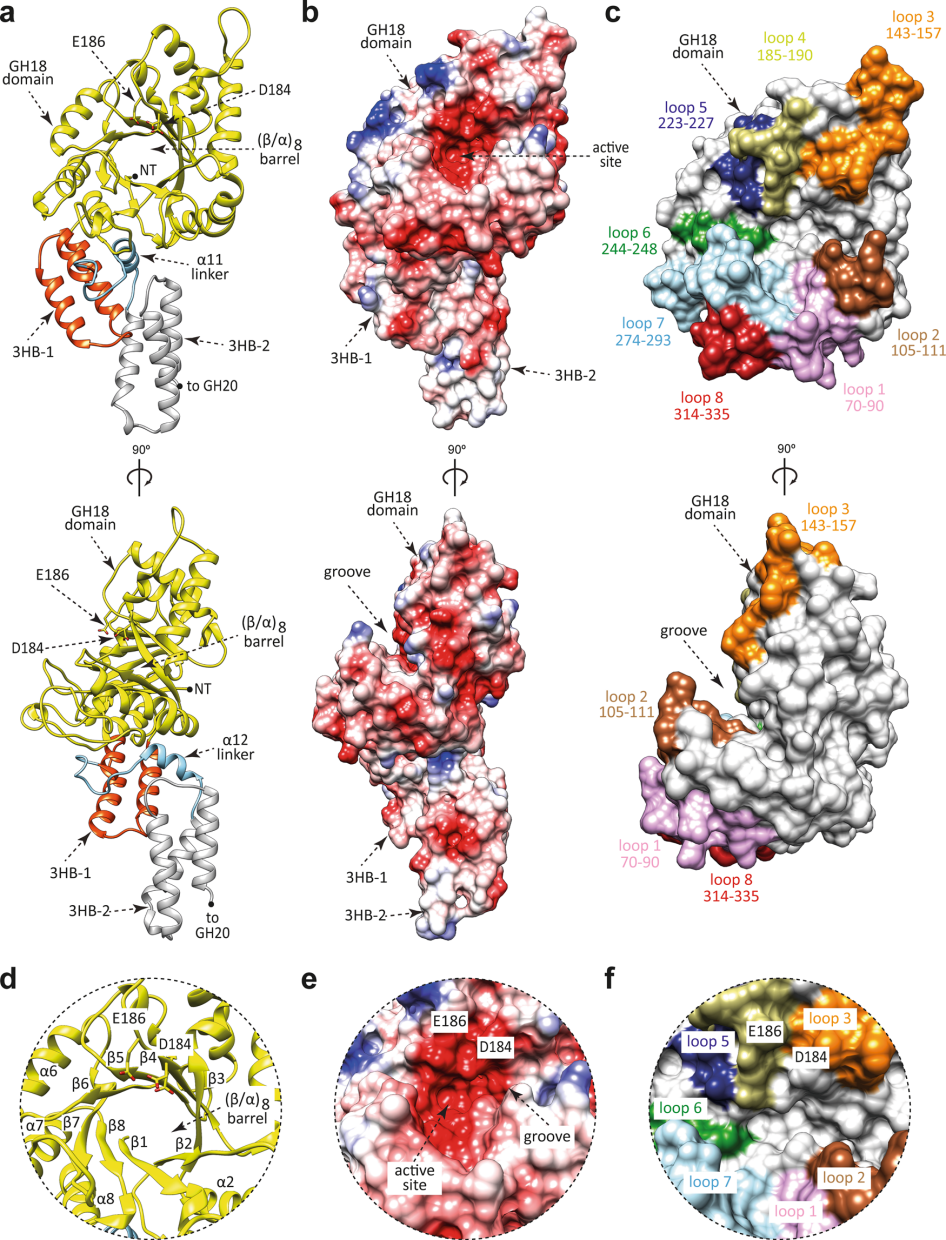
The overall structure of the GH18 domain of EndoE. **a** Two cartoon representations showing the general fold and secondary structure organization of EndoE-GH18L, including the GH18 domain (yellow), the 3HB-1 (dark orange), and 3HB-2 (light gray) domains, and the α12 linker (light blue). **b** Two electrostatic surface representations of EndoE-GH18L showing the location of the putative *N*-glycan binding site and the catalytic site. **c** Two surface representations of the GH18 domain of EndoE, with annotated domains and loops. **d** Close-up view of the active site of the GH18 domain of EndoE shown as cartoon/stick representation (yellow). **e** Close-up view of the active site of the GH18 domain of EndoE shown as electrostatic surface representation. **f** Close-up view of the active site of the GH18 domain of EndoE shown with annotated loops.

EndoE is a multi-modular enzyme that includes protein regions, from N- to C-terminus, as follows: (i) a GH18 domain, (ii) a ‘linker region’, rich in predicted α-helices, and (iii) a GH20 domain. Therefore, we generated four truncated constructs of full-length EndoE (EndoE; residues 56–837; residues 1–55 correspond to the signal peptide)^18^ that contained one of the glycoside hydrolase domains either with or without the linker region: EndoE-GH18 (residues 56–370; GH18 domain), EndoE-GH18L (residues 56–486; GH18 domain and ‘linker region’), EndoE-LGH20 (residues 371–837; ‘linker region’ and GH20 domain), and EndoE-GH20 (residues 487–837; GH20 domain; Supplementary Fig. 1a). We crystallized the EndoE-GH18L and EndoE-GH20 constructs. We solved the crystal structure of EndoE-GH18L by molecular replacement method (PDB code 7PUJ; Fig. 2; Supplementary Figs. 1 and 2; Supplementary Table 1 and Methods section). EndoE-GH18L crystallized in the *P* 6 5 space group with one molecule in the asymmetric unit and diffracted to a maximum resolution of 1.7 Å (Supplementary Table 1). The high quality of the electron density maps allowed us to trace residues 61–482. The GH18 domain (residues 61–349) adopts the conserved (β/α)_8_-barrel topology with an overall size of 45 × 46 × 38 Å (Fig. 2a, d). A large groove contains the active site (Fig. 2c, f). This groove is located at the center of the (β/α)_8_-barrel and surrounded by the loops connecting β1–α1 (loop 1; residues 70–90, pink), β2–α2 (loop 2; residues 105–111, brown), β3–α3 (loop 3; residues 143–157, orange), β4–α4 (loop 4; residues 185–190, light green), β5–α5 (loop 5; residues 223–227, blue), β6–α6 (loop 6; residues 244–248, green), β7–α7 (loop 7; residues 274–293, light blue), and β8–α8 (loop 8; residues 314–335, red) (Fig. 2a, d). The floor of this groove is composed of a set of aromatic residues including Y67, W71, F104, Y245, and Y313. We propose that EndoE GH18 follows a two-step substrate-assisted catalytic mechanism, as previously described for other GH18 family members^37–44^. In the first step, the *N*-linked glycan substrate binds to the active site inducing the distortion of the GlcNAc (-1). In this step, the E186 residue (loop 4) protonates the glycosidic bond, acting as an acid, whereas the D184 residue (β4) orients the oxygen of the C2-acetamide group of GlcNAc (-1), which attacks the anomeric carbon of GlcNAc (-1) and leads to the formation of an oxazolinium ion intermediate. In the second step, E186 acts as a base, deprotonating a water molecule that performs a second nucleophilic attack and breaks the oxazoline ring, regenerating the hemiacetal sugar with retention of anomeric configuration (Supplementary Fig. 3a).

A search for structural homologues using the DALI server^45^ (see Methods section) revealed glycoside hydrolases with significant structural similarity to the GH18 domain of EndoE (Supplementary Fig. 4a), including: (i) Endo-CoM from *Cordyceps militaris* (PDB code 6KPO; Z-score of 32.7; r.m.s.d. value of 2.0 Å for 262 aligned residues; 32% identity), an enzyme that is active exclusively on α(1, 3) fucosylated biantennary CT *N*-glycans^46^, (ii) EndoS from *S. pyogenes* (PDB code 4NUY; Z-score of 30.6; r.m.s.d. value of 2.5 Å for 268 aligned residues, 26% identity), and (iii) EndoS2 from *S. pyogenes* (PDB code 6E58; Z-score of 30.5; r.m.s.d. value of 2.6 Å for 269 aligned residues, 26% identity).

### Structure of the linker region of EndoE

The crystal structure of the EndoE-GH18L construct revealed that the linker region folds into two three α-helical bundle (3HB) domains connected by an additional α-helix (Supplementary Fig. 5)^47^. The first 3HB domain comprises helices α9, α10 and α11 (3HB-1; residues 350–395), whereas the second 3HB domain covers helices α13, α14, and α15 (3HB-2; residues 421–482). Both 3HB-1 and 3HB-2 domains are connected by a short α-helix, α12 (residues 409–417; Supplementary Fig. 5a). The α12 helix participates in the interaction between the GH18, 3HB-1, and 3HB-2. The GH18 and 3HB-1 domains interact with each other through an extensive contact area of ca. 972 Å^2^, representing 7% of the total accessible surface of the isolated domains (Supplementary Fig. 5b)^48^. The interface is mainly mediated by: (i) α7, α8, loops 7 and 8 of the GH18 domain; and (ii) α11 of the 3HB-1, supporting the notion of a stable association between both domains (Supplementary Fig. 5d, e). Y388 from 3HB-1 forms a hydrophobic interaction with the side chains of Y290 and W337 from GH18 domain, while D398 from short α-helix α12 and K392 from 3HB-1 form salt bridges with R320 and E288 from the GH18 domain, respectively, and Y399 from α-helix α12 establish hydrophobic interactions with P332 from 3HB-1. Furthermore, the 3HB-1 and 3HB-2 domains mainly interact with each other through the C-terminal and N-terminal tips, respectively (Supplementary Fig. 5f). H359 and Y366 from 3HB-1 make hydrogen bonds with the side chains of Y464 and D428 from 3HB-2, respectively. R363 from 3HB-1 also establishes a hydrogen bond with the main chain of G461 from 3HB-2. Y424 from 3HB-2 interacts through a hydrogen bond with the main chain of R363 and through hydrophobic interaction with the side chain of K362 from 3HB-1.

The 3HB-2 domain displays low structural homology with: (i) the C-terminal 3HB domain of EndoS from *Streptococcus pyogenes* (PDB code 4NUZ; Z-score of 10.4; r.m.s.d. value of 3.2 Å for 83 aligned residues, 11% identity), for which no function was reported and is dispensable for the enzymatic activity^49^; and (ii) the FIVAR (Found-In-Various Architectures) module of NagH from *Clostridium perfringens* (PDB code 2OZN; Z-score of 9.5; r.m.s.d. value of 1.6 Å for 64 aligned residues; 13% identity; NagH is a carbohydrate-active μ-toxin with hyaluronidase activity that contains a GH84 domain, along with several CBM and FIVAR modules^50^); and (iii) the three-helix bundle motif of alpha C protein from *Streptococcus agalactiae* (PDB code 1YWM; Z-score of 9.3; r.m.s.d. value of 4 Å for 80 aligned residues; 15% identity; Supplementary Fig. 4b). Alpha C protein is an invasin that participates in the translocation of group B *Streptococcus* across human epithelial cells. It contains a three-helix bundle involved in the formation of a potential heparin-binding site^51^.

### Structure of the GH20 domain of EndoE

**Fig. 3:**
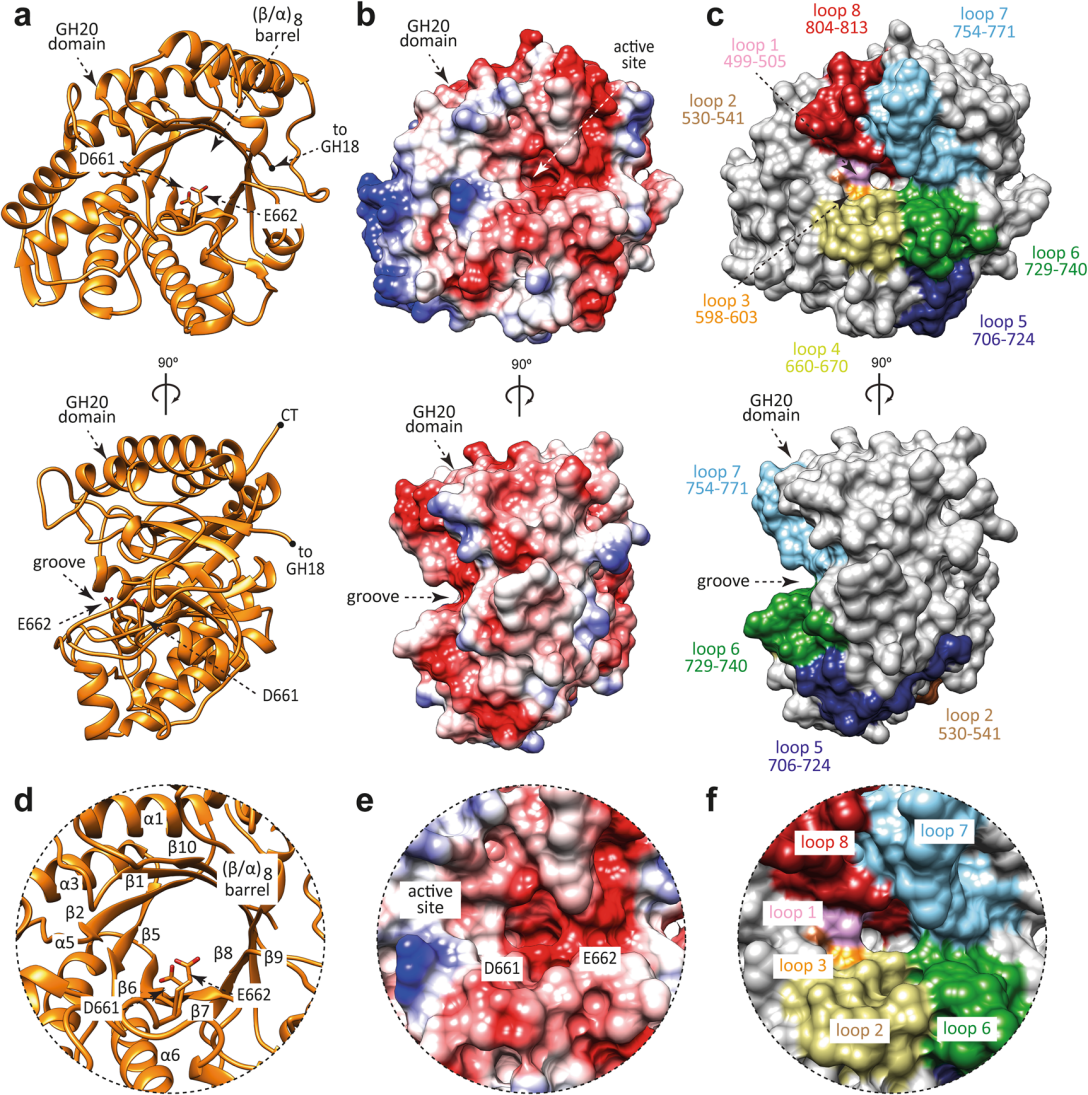
The overall structure of the GH20 domain of EndoE. **a** Two cartoon representations showing the general fold and secondary structure organization of EndoE-GH20 (orange). **b** Two electrostatic surface representations of EndoE-GH20 showing the location of the putative *N*-glycan binding site and the catalytic site. **c** Two surface representations of the GH20 domain of EndoE, with annotated domains and loops. **d** Close-up view of the active site of the GH20 domain of EndoE shown as cartoon/stick representation (orange). **e** Close-up view of the active site of the GH20 domain of EndoE shown as electrostatic surface representation. **f** Close-up view of the active site of the GH20 domain of EndoE shown with annotated loops.

We solved the crystal structure of EndoE-GH20 by molecular replacement methods (PDB code 7PUL; Fig. 3; Supplementary Figs. 1 and 2; Supplementary Table 1 and Methods section). EndoE-GH20 crystallized in the *P* 1 2 1 1 space group with one molecule in the asymmetric unit and diffracted to a maximum resolution of 1.4 Å (Supplementary Table 1). The quality of the electron density maps allowed us to trace residues 489–835. The GH20 domain adopts the conserved (β/α)_8_-barrel topology with an overall size of 55 × 50 × 39 Å (Fig. 3a, d). A long and open groove, including a deep pocket, runs parallel to the protein surface and contains the active site (Fig. 3b, c). This groove is located at the center of the (β/α)_8_-barrel and surrounded by the loops connecting β1-α1 (loop 1; residues 499–505, pink), β2-β3 (loop 2; residues 530–541, brown), β5-α4 (loop 3; residues 598–603, orange), β6-α6 (loop 4; residues 660–670, light green), β7-β8 (loop 5; residues 706–724, blue), β8-α7 (loop 6; residues 729–740, green), β9-α8 (loop 7; residues 754–771, light blue), and β10-α9 (loop 8; residues 804–813, red; Fig. 3c). The deep pocket is primarily composed of a set of aromatic residues including H602, F705, W729, and W804.

A structural homology search using the DALI server^45^ revealed only one protein with high structural similarity to the GH20 domain of EndoE, that of StrH from *Streptococcus pneumoniae* (Supplementary Fig. 4c). Coincidentally, StrH contains two catalytic domains that belong to the GH20 family, GH20A (PDB code 2YL8; Z-score of 57.0; r.m.s.d. value of 0.8 Å for 346 aligned residues; 53% identity) and GH20B (PDB code 2YLA; Z-score of 56.3; r.m.s.d. value of 0.9 Å for 345 aligned residues; 49% identity). StrH is able to hydrolyze the β(1,2) linkages between the non-reducing terminal GlcNAc to a Man residue in CT *N*-glycans^52–54^. The high structural similarity suggests that the GH20 domain of EndoE also has exo-β-*N*-acetylglucosaminidase activity. In addition, structural comparison of GH20A and GH20B domains of StrH with the GH20 domain of EndoE reveals the conservation of the two catalytic residues in loop 4 that mediate a substrate-assisted mechanism similar to that described for the GH18 domain^55,56^. In a first step, D661 stabilizes the reaction intermediate and orients the acetamido oxygen for nucleophilic attack on the anomeric carbon to form an oxazolium ion intermediate. In addition, E662 acts as an acid protonating the glycosidic bond. In contrast, in a second step, E662 acts as a base, deprotonating a water molecule that hydrolyze the oxazolium ion intermediate to yield a product with the same anomeric center as the substrate (Supplementary Fig. 3)^55,56^.

Two other GH20 enzymes displayed low structural homology to the GH20 domain of EndoE: (i) dispersin B from *Actinobacillus actinomycetemcomitans* (PDB code 1YHT; Z-score of 31.8; r.m.s.d. value of 2.8 Å for 298 aligned residues; 20% identity); and (ii) lacto-*N*-biosidase (LNBase) from *Bifidobacterium bifidum* (PDB code 5BXR; Z-score of 31.0; r.m.s.d. value of 2.5 Å for 290 aligned residues; 22% identity; Supplementary Fig. 4c). Dispersin B is a soluble exo-β-*N*-acetylglucosaminidase that degrades poly-β-1,6-*N*-acetyl-D-glucosamine (PNAG), a major component of the polysaccharide matrix that constitutes the biofilms formed by many bacteria^57^. Structural comparison of GH20 domain of EndoE with dispersin B revealed that loops 4 and 6 adopt a unique conformation that could explain the different substrate linkage accepted by each of these enzymes. LNBase from *B. bifidum* is a key enzyme that hydrolyzes lacto-*N*-tetraose (Gal*β*1 − 3GlcNAc*β*1 − 3Gal*β*1 − 4Glc), the main component of human milk oligosaccharides, to lead lacto-*N*-biose I (Gal*β*1 − 3GlcNAc*β*1) and lactose (3Gal*β*1 − 4Glc)^58^. Structural comparison of GH20 domain of EndoE with LNBase showed that the conformation and length of loops 1 and 8 in EndoE-GH20 could block the entrance of an extra carbohydrate moiety linked to GlcNAc (-1), confirming the exo-activity of this domain.

### Structure of full-length EndoE

**Fig. 4:**
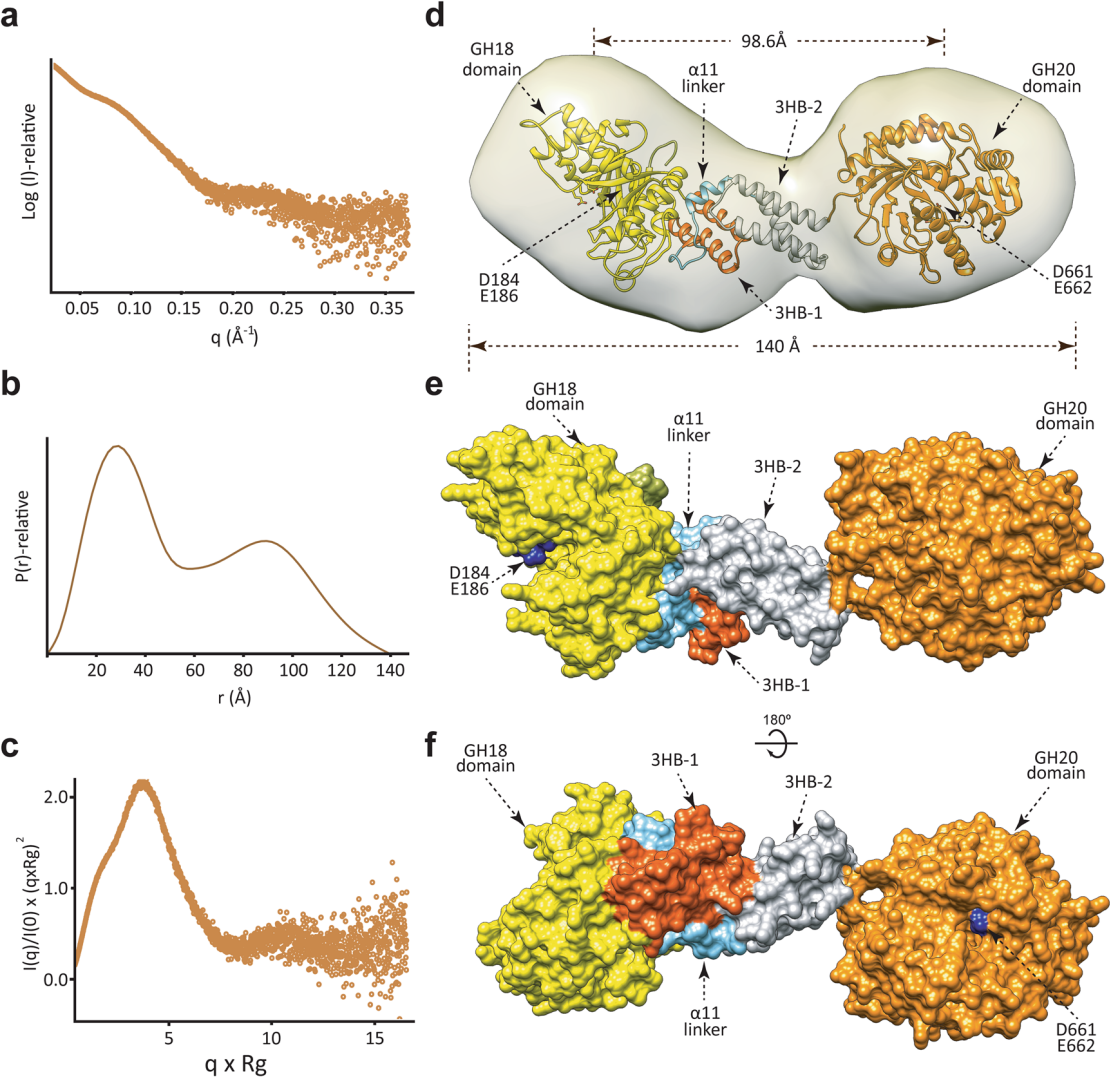
The overall structure of the full-length EndoE. **a** SAXS scattering curve of full-length EndoE. **b** *P(r)* functions distributions of full-length EndoE. **c** Normalized Kratky plot of full-length EndoE. **d** Ab initio SAXS envelope of full-length EndoE using GASBOR. Superposition of the model of full-length EndoE. The GH18, linker, and GH20 domains are colored in yellow, green, and orange, respectively. **e**, **f** Two views of the surface representation of EndoE, showing the architecture of the GH18 (yellow), 3HB-1 (dark orange), 3HB-2 (gray), and GH20 (light orange) domains.

To study the overall structure of EndoE in solution and the arrangement of each domain, we performed in-line size-exclusion chromatography SAXS (SEC-SAXS; Fig. 4; Supplementary Table 2; Methods). SAXS is a powerful technique capable of providing structural information on flexible and dynamic proteins in solution^59^. EndoE elutes from the gel filtration column as a monomer with an average MW of 90 kDa (88.3 kDa is the expected MW of the EndoE sequence; Supplementary Fig. 1) and a radius of gyration, *R*_*g*_, an average of square center-of-mass distances in the molecule weighted by the scattering length density, of 46.5 Å (Fig. 4c). The interatomic distribution function *P(r)* is a measure of the frequency of interatomic vector lengths within a protein molecule that provides information about the shape of the scattering particle. The resulting *P(r)* function profile exhibits a bimodal distribution of real-space distances, indicating that the particle is elongated. The maximal diameter *D*_*max*_ is ca. 140 Å, with a maximum peak at r = 30 Å and a secondary peak at 90 Å (Fig. 4b). The SAXS results are summarized in Fig. 4, and Supplementary Table 2.

We reconstructed the ab initio low-resolution envelope of EndoE using GASBOR (Supplementary Table 2). As depicted in Fig. 4d-f, the envelope of EndoE displays a ‘bean’ shape with two asymmetrical lobes. The dimension of EndoE is ca. 141 × 48 × 42 Å, while the dimensions of the individual big and small lobes are ca. 68 × 48 × 42 Å and ca. 61 × 43 × 42 Å, respectively (Fig. 4e). Our crystal structures of the EndoE-GH18L and EndoE-GH20 constructs clearly show that EndoE comprises four domains from the N- to the C-terminus: (i) a GH18 domain (residues 61–349), (ii) a 3HB-1 domain (residues 350–395), (iii) a 3HB-2 domain (residues 421–482), and a GH20 domain (residues 487–837). Therefore, we fit EndoE into the ab initio low-resolution envelope with the EndoE-GH18L and GH20 structures occupying the big and small lobes, respectively (Fig. 4d). Supporting this model, the GH18 domain is well-packed against the ‘linker region’, including the 3HB-1 and 3HB-2 domains, whereas the GH20 domain is linked to ‘linker region’ through a small loop (residues 485–491), suggesting that this domain might be flexible to facilitate access to substrates. Consequently, the catalytic residues of the GH18 (D184 and E186) and the GH20 (D661 and E662) domains are 98.6 Å apart from each other, and are on opposite sides of the full-length enzyme. The structural arrangement of the EndoE catalytic domains may facilitate access to a diverse set of substrates.

### Substrate specificities of EndoE and its constitutive GH18 and GH20 domains

To further investigate the role of each of the two glycoside hydrolase domains in the EndoE substrate specificity mechanism, we performed enzymatic activity assays with EndoE and the individual GH18 and GH20 domains against: (i) Rituximab, a chimeric therapeutic monoclonal antibody bearing a human IgG1 Fc region containing mostly CT *N*-glycans attached to N297 of both heavy chains (Supplementary Fig. 6a); (ii) RNAse B, an endoribonuclease that exhibits a single *N*-linked glycosylation site at N34 bearing HM-type *N*-glycans; (iii) RNAse A, the non-glycosylated version of RNAse B, as a negative control (Supplementary Fig. 6b) and (iv) transferrin, a protein which contains sialylated bi- and tri- antennary CT *N*-glycans (Supplementary Fig. 7 and Supplementary Table 4). Specifically, Rituximab, RNAse B, RNAse A and transferrin were incubated in the presence of either the purified EndoE, EndoE-GH18L, EndoE-GH20, or a mixture of EndoE-GH18L/EndoE-GH20 constructs. As depicted in Supplementary Fig. 6c, EndoE was able to process Rituximab. However, neither EndoE-GH18L nor EndoE-GH20 constructs hydrolyzed the *N*-glycan from Rituximab. We found that the activity of EndoE against Rituximab was restored when we incubated a mixture of EndoE-GH18L and EndoE-GH20 with Rituximab (Supplementary Fig. 6c). EndoE was able to process *N*-glycans from RNAse B (Supplementary Fig. 6d). This activity is due to the presence of EndoE-GH18L since EndoE-GH20 is not active on RNAse B (Supplementary Fig. 6d). Taken together, these data suggest that EndoE is capable of hydrolyzing CT and HM-type *N*-glycans from Rituximab and RNAse B, respectively. The GH18 domain of EndoE is required for both ENGase activities. However, to process CT *N*-glycans, the GH18 domain first requires the action of the GH20 domain, likely an exo-β-*N*-acetylglucosaminidase, to trim the *N*-glycan.

**Fig. 5:**
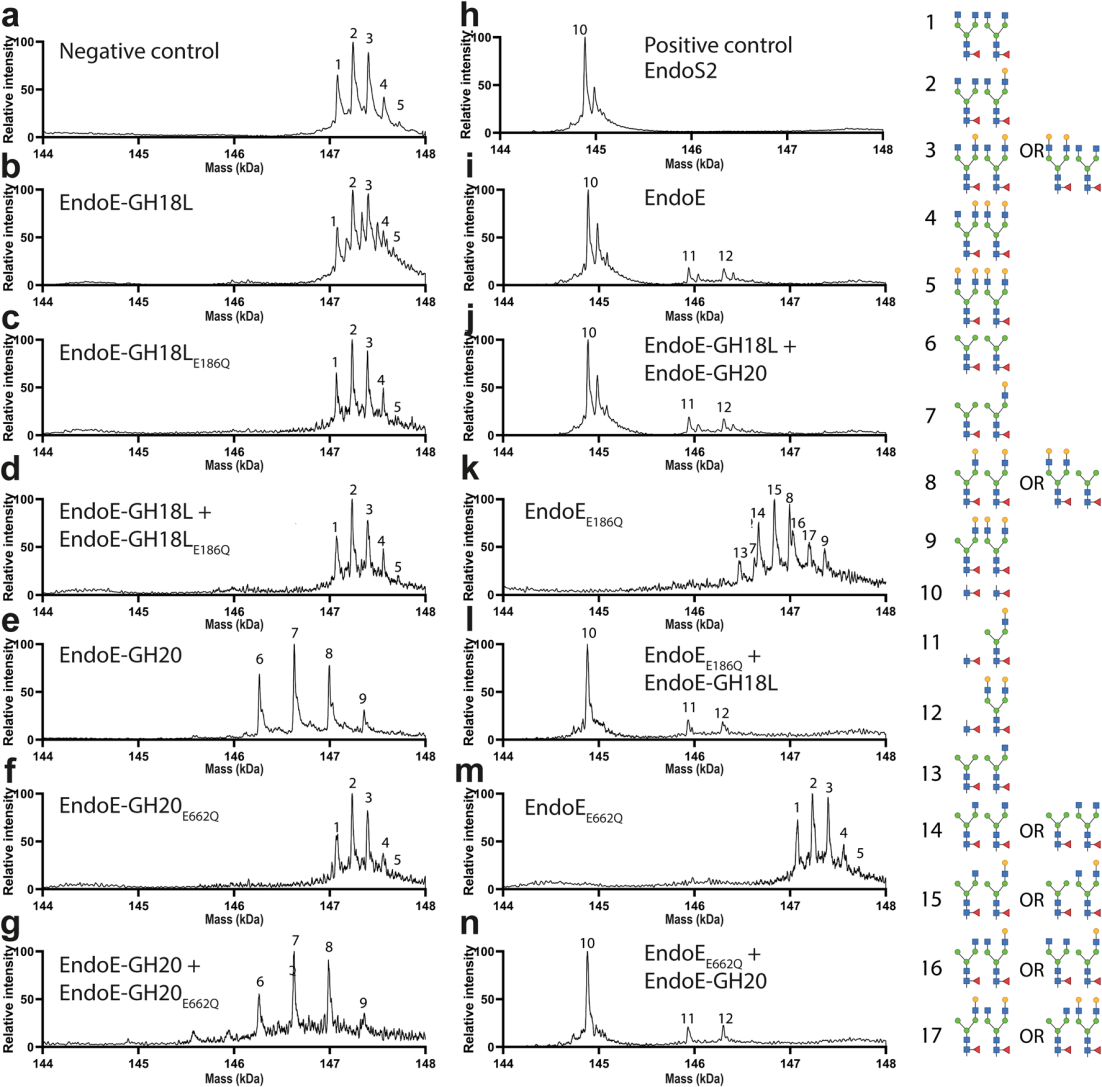
Molecular basis of EndoE GH18 and GH20 domains substrate specificity as visualized by LC/MS. Processing of CT *N*-glycans on Rituximab. Mass spectrometry of Rituximab treated with **a** no enzyme (negative control) **b** EndoE-GH18L **c** EndoE-GH18L_E186Q_ **d** EndoE-GH18L + EndoE-GH18L_E186Q_ **e** EndoE-GH20 **f** EndoE-GH20_E662Q_ **g** EndoE-GH20 + EndoE-GH20_E662Q_ **h** EndoS2 (positive control) **i** EndoE **j** EndoE-GH18L + EndoE-GH20 **k** EndoE_E186Q_ **l** EndoE_E186Q_ + EndoE-GH18L **m** EndoE_E662Q_ **n** EndoE_E662Q_ + EndoE-GH20. The peaks corresponding to intact Rituximab are numbered based on the glycoforms found in each heavy chain. The retention time for Rituximab was 2.4 min. For mass deconvolution, the following parameters were used in the BioConfirm software; 2000–7000 *m/z* and 14.4–14.8 kDa. The theoretical and observed mass of each annotated peak are in Supplementary Table 4.

**Fig. 6:**
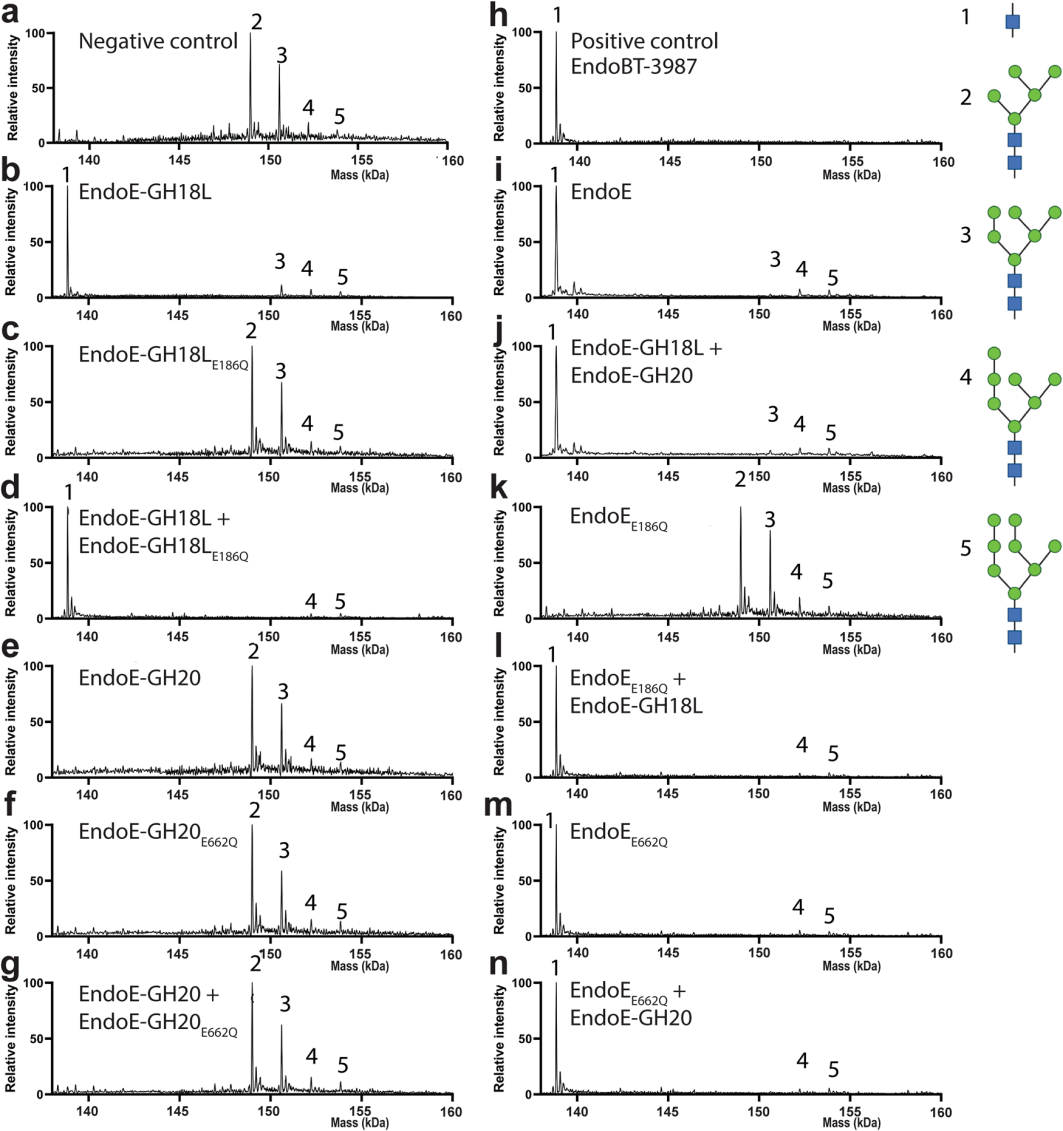
Molecular basis of EndoE GH18 and GH20 domains substrate specificity as visualized by LC/MS. Processing of HM-type *N*-glycans on RNAse B. Mass spectrometry of RNAse B treated with **a** no enzyme (negative control) **b** EndoE-GH18L **c** EndoE-GH18L_E186Q_ **d** EndoE-GH18L + EndoE-GH18L_E186Q_ **e** EndoE-GH20 **f** EndoE-GH20_E662Q_ **g** EndoE-GH20 + EndoE-GH20_E662Q_ **h** EndoBT-3987 (positive control) **i** EndoE **j** EndoE-GH18L + EndoE-GH20 **k** EndoE_E186Q_ **l** EndoE_E186Q_ + EndoE-GH18L **m** EndoE_E662Q_ **n** EndoE_E662Q_ + EndoE-GH20. The peaks corresponding to intact RNaseB are numbered based on the glycoforms found in the single glycosylation site of the protein. The retention time for RNAseB was 2.1 min. For mass deconvolution, the following parameters were used in the BioConfirm software; 1000–2400 *m/z* and 130–160 kDa. The theoretical and observed mass of each annotated peak are in Supplementary Table 4.

We performed LC-MS analysis to further investigate the activity of EndoE and its individual domains. Specifically, Rituximab and RNAse B were incubated with either active or inactive forms of EndoE, EndoE-GH18L or EndoE-GH20 domains (Figs. 5 and 6 and Supplementary Table 4). In cases where the inactive form was incubated with either Rituximab or RNAse B, we also performed a similar experiment where the active form of the original inactive enzyme was also included in the reaction mixture. The results of LC-MS analysis of Rituximab processing by EndoE is shown in Fig. 5. EndoE-GH18L was unable to process any of the glycans on Rituximab (Fig. 5b) while EndoE-GH20 domain only removed terminal GlcNAc moieties (Fig. 5e), such as those present on glycoforms with no terminal Gal in any antennae (G0) and a single terminal Gal in the α(1,3) or α(1,6) antennae (G1), resulting in the formation of 4 glycoforms (6, 7, 8, and 9) which contain one or two terminal galactoses and no terminal GlcNAc. As expected, the inactive domains of EndoE produced by mutation of the acid/base residue that participates in the hydrolysis of the glycosidic bond by glutamine, E186Q and E662Q in GH18 and GH20 domain, respectively, exhibited no hydrolytic activity against Rituximab (Fig. 5c, f). EndoE was able to hydrolyze some of the glycans from Rituximab (Fig. 5i) and the same glycoforms were found after treatment of Rituximab with the individual domains GH18 and GH20 (Fig. 5j). The presence of glycoforms (11) and (12) suggest that the GH18 domain was not capable of releasing neither certain G1 glycans nor G2 glycans from Rituximab. Incubation of EndoE_E186Q_ with Rituximab (Fig. 5k) resulted in the formation of extra glycoforms (13, 14, 15, 16, and 17) compared to processing by EndoE-GH20 alone. These glycoforms contain terminal GlcNAc moieties, suggesting that the presence of a linked GH18 domain attenuates its activity. However, incubation of EndoE_E186Q_, GH18 domain, and Rituximab produced the same glycoforms as with EndoE, suggesting that the GH18 domain could be acting on Man_3_GlcNAc_2_ (Man_3_), as well as CT glycoforms with a terminal GlcNAc, such as (13) (Fig. 5l). To further investigate this, we also incubated Rituximab treated with BgaA galactosidase (producing Rituximab with only the G0/G0 glycoforms (1)) with active forms of EndoE, EndoE-GH18L, or EndoE-GH20 (Supplementary Fig. 8 and Supplementary Table 4). The results showed that the GH20 and GH18 domains work in concert to produce fully deglycosylated Rituximab by removal of the terminal GlcNAc saccharide and Man_3_ glycans, respectively.

To investigate the role of the linker in EndoE, we also carried out a kinetic analysis of glycan hydrolysis by LC-MS. We incubated either EndoE or EndoE-GH18L + EndoE-GH20 in a 1:1 stoichiometric ratio, and Rituximab and tracked the rate of formation of monoglycosylated and deglycosylated Rituximab (Supplementary Fig. 12). The rate of release of the first glycan was two-fold faster for EndoE compared to the individual domains (0.45 and 0.20 nM s^−1^ for EndoE and EndoE-GH18L + EndoE-GH20, respectively). The rate of release for the second glycan was ten-fold faster for EndoE compared to the individual domains (2.22 and 0.19 nM s^−1^ for EndoE and EndoE-GH18L + EndoE-GH20, respectively). In combination with other data presented, this experiment shows that the GH20 domain is required for the full enzymatic activity of EndoE, irrespective of whether the domain is fused to the GH18 domain. The differing rates between EndoE and EndoE-GH18L + EndoE-GH20 suggest that the linker allows the two domains to work synergistically, especially in complex environments, such as the human gut environment. The activity of EndoE on RNAse B is shown in Fig. 6. As depicted in Fig. 6b, GH18 can release Man_5_ and Man_6_ glycans from RNAse B and may also be capable of releasing Man_7_ at a much slower rate. GH20 has no effect on RNAse B (Fig. 6e), indicating that it has no activity on mannose.

**Fig. 7:**
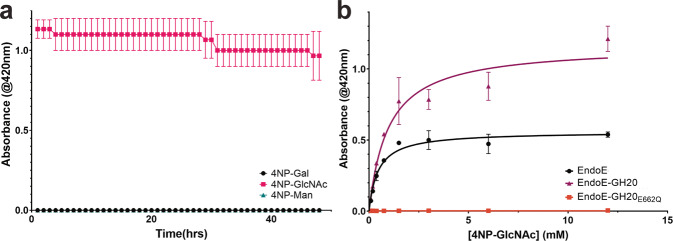
Kinetic analysis of EndoE-GH20 domain substrate specificity. **a** Determination of EndoE-GH20 domain substrate specificity by 4-nitrophenol (4NP) linked substrates. **b** Kinetic analyses of GH20 domain as part of EndoE or alone. The data plotted is the mean of three independent experiments (n), with the standard deviation (SD) being plotted as the error. Source data are provided as a Source Data file.

To confirm our hypothesis that the GH20 domain of EndoE exhibits exo-β-*N*-acetylglucosaminidase activity, we also performed activity assays with 4-nitrophenol-linked galactose, β-*N*-acetylglucosamine, and mannose. To test the substrate specificity of GH20, we incubated each of these substrates with EndoE or EndoE-GH20. As shown in Fig. 7a, GH20 exhibited only exo-β-*N*-acetylglucosaminidase activity. We also performed kinetic analyses to determine the catalytic efficiency of the GH20 domain (Fig. 7b), both as part of the holo-enzyme and as an individual domain. We determined the turnover rate (*k*_cat_) of the GH20 as part of EndoE to be 41 s^−1^ and a *K*_m_ = 0.45 mM yielding a *k*_cat_/*K*_m_ of ~92 s^−1^ mM^−1^. Similar analysis for the GH20 domain alone yielded *k*_cat_ = 86 s^−1^ and *K*_m_ = 0.97mM, yielding a *k*_cat_/*K*_m_ of ~89 s^−1^ mM^−1^. Altogether, the activity measurements support the notion that the GH18 and GH20 domains work in concert to process *N*-glycans. The GH20 domain exhibits exo-β-*N*-acetylglucosaminidase activity to produce shorter glycans that can be processed subsequently by the GH18 domain.

### Structural basis of EndoE GH18 and GH20 domain substrate specificity

**Fig. 8:**
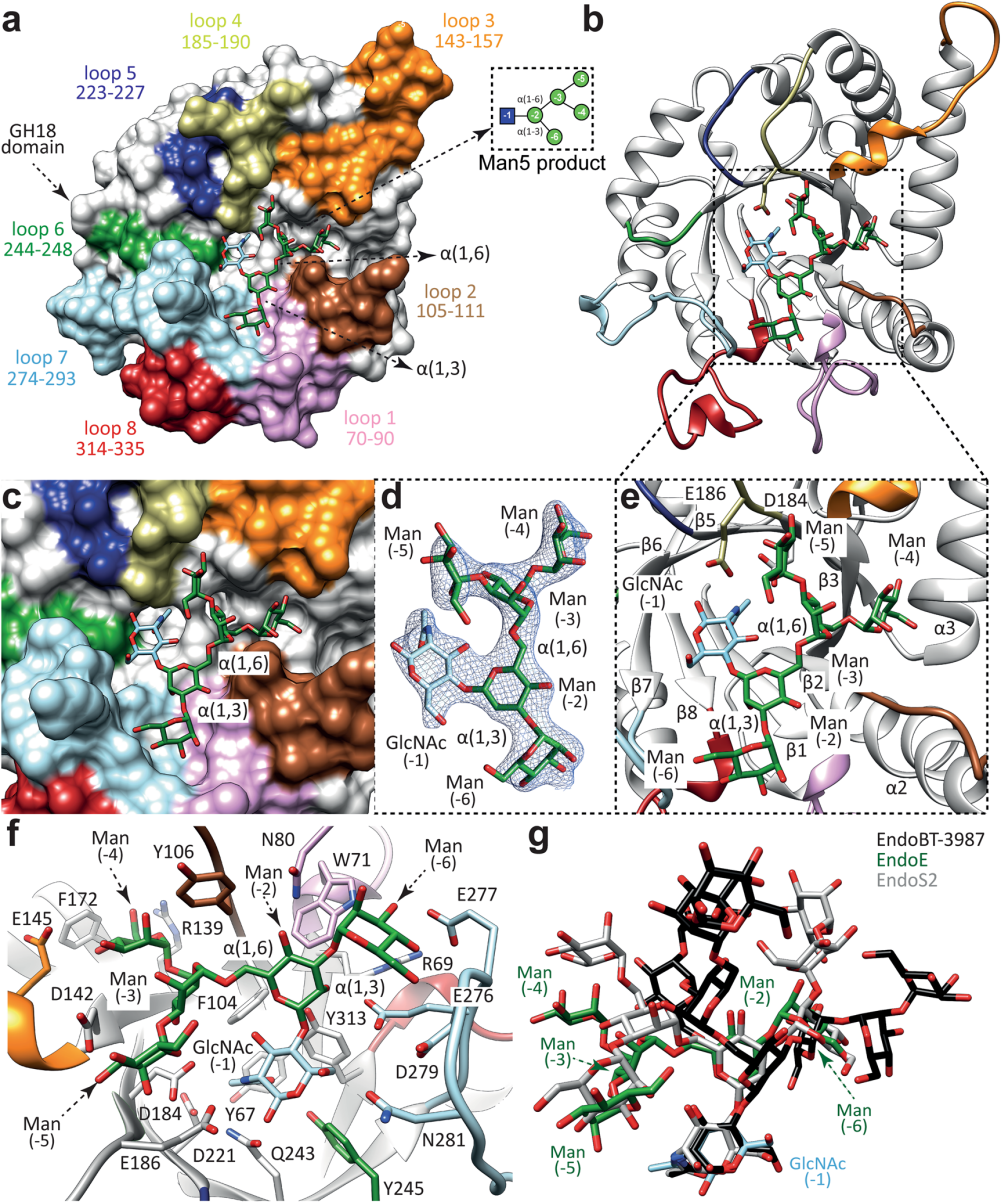
Structural basis of EndoE GH18 domain substrate specificity. **a** Surface representation of the EndoE-GH18L-Man_5_ crystal structure, with annotated domains and loops. **b** Cartoon representation of the EndoE-GH18L-Man_5_ crystal structure. **c** Surface representation of the GH18 domain of EndoE showing the location of the Man_5_ product into the active site. **d** Electron density map of the Man_5_ product shown at 1.0 σ r.m.s.d. **e** Cartoon representation of the GH18 domain of EndoE showing the location of the Man_5_ product into the active site. **f** Cartoon representation of the GH18 domain of EndoE showing the main residues and secondary structure elements interacting with the Man5 product in the active site. **g** Superposition of the HM-type *N*-glycan found in the X-ray crystal structures of EndoE-GH18L (green), EndoBT-3987 (black), and EndoS2 (gray).

To better understand the molecular mechanisms by which the GH18 and GH20 domains of EndoE process-specific *N*-glycans, we first solved the crystal structure of EndoE-GH18L in complex with the Man_5_GlcNAc (Man_5_) product (EndoE-GH18L-Man_5_; Fig. 8; PDB code 7PUK; Supplementary Figs. 1 and 2; Supplementary Table 1 and Methods section). EndoE-GH18L-Man_5_ crystallized in the *C* 1 2 1 space group with two molecules in the asymmetric unit and diffracted to a maximum resolution of 2.6 Å (Supplementary Table 1). The structural comparison of EndoE-GH18L and the product bound EndoE-GH18L-Man_5_ revealed that the protein structure is mostly preserved upon glycan binding and that there are no substantial conformational changes (r.m.s.d. of 0.58 Å for 416 residues). The Man_5_ product could be modelled unambiguously in the electron density map, located at the center of the (β/α)_8_-barrel and surrounded by the loops 1 to 8 (Figs. 2 and 8a-e). The overall conformation of the Man_5_ product is equivalent in the two molecules of the asymmetric unit (r.m.s.d. of 0.45 Å)^60^. The GlcNAc (-1) residue adopts a chair conformation (^4^*C*_1_) in which O1 makes hydrogen bonds with the side chains of E186 and Q243, and O3 establishes an intramolecular hydrogen bond with the cyclic oxygen atom in the Man (-2) ring (Fig. 8b-e). The O4 and O6 make hydrogen bonds with the side chains of Y313 and N281, respectively. The N2 atom of the acetamide group interacts through hydrogen bonds with the side chains of D184 and E186, whereas the O7 atom makes a hydrogen bond with the side chain of Y245 (Fig. 8b-e). This conformation likely corresponds to a stage of the catalytic cycle of the enzyme shortly before the release of the glycan product^38,61^. The O2 of Man (-2) makes hydrogen bonds with the side chains of E276 and Y313, and so does O4 with the side chain of N80. The O4 of Man (-3) makes a hydrogen bond with the side chain of D184 (Fig. 8b-e). The O4 and O5 of Man (-4) interact, respectively, with the side chains of E145 and R139 through a hydrogen bond. The O2 of Man (-5) makes a hydrogen bond with the side chain of D184, and O3 makes a hydrogen bond with the side chain of D142. Finally, O2 and O6 of Man (-6) make hydrogen bonds with the main chains of N80 and E277, respectively. O3 makes hydrogen bonds with the side chains of W71 and E277 and O4 establishes a hydrogen bond with the side chain of R69 (Fig. 8b-e).

The crystal structure of the EndoE-GH18L-Man_5_ complex reveals that the Man_5_ glycan is accommodated in a groove that forms a narrow space for the α(1,3) antenna. Specifically, loop 1 of the GH18 domain clearly restricts the access of additional Man residues with respect to Man (-6). In line with these results, molecular docking calculations placed Man_6_ in an energetically favorable position (estimated affinity of −20.8 kcal mol^−1^)^62^ where the α(1,6) antenna retains the position of the crystallographic Man_5_ conformation while the α(1,3) antenna is slightly moved away from the protein groove to correctly fit Man (-7) (Supplementary Figs. 9 and 10). Conversely, molecular docking calculations for the Man_7_ glycan containing the three Man residues of the α(1,3) antenna, Man (-6), Man (-7), and Man (-8), place the glycan into an energetically unfavorable conformation (estimated affinity of 21.9 kcal mol^−1^)^62^ in which GlcNAc (-1), Man (-6) and Man (-8) have significant clashes with the protein. Furthermore, molecular docking calculations for the Man_9_ *N*-glycan containing two additional α(1,2)-mannoses connected to Man (-6) and Man (-4) residues of the α(1,6) antenna also show major clashes with loop 1,2 and 4 of the protein (Supplementary Fig. 9). Our hydrolytic activity measurements showed that the GH18 domain is only capable of hydrolyzing CT *N*-glycans after the action of the GH20 domain, which hydrolyzes the terminal GlcNAc residues. Our molecular docking calculations with CT *N*-glycans of different sizes show that the GH18 domain can accommodate CT *N*-glycans with a terminal GlcNAc (-4) in the α(1,6) antenna at the binding site while binding CT *N*-glycan with a terminal GlcNAc (-6) of α(1,3) antenna would be unfavorable due to steric hindrance between this carbohydrate residue and loop 1. Similarly, CT *N*-glycans capped with Gal residues and hybrid type (Hy-type) *N*-glycans could also accommodate the α(1,6) but not the α(1,3) antenna.

**Fig. 9:**
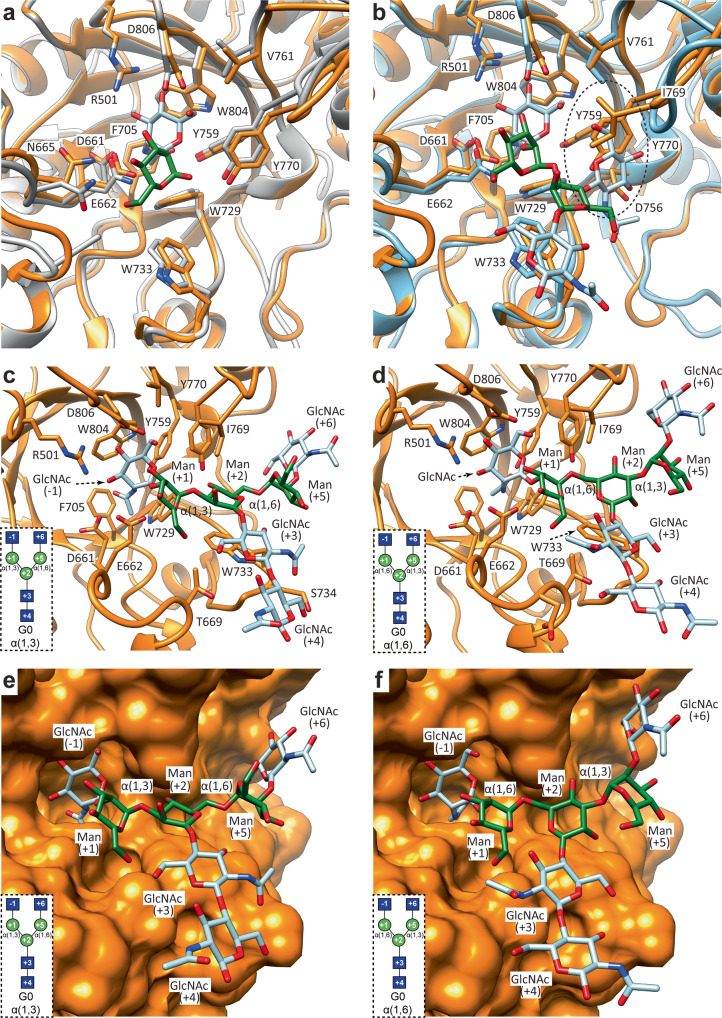
Structural basis of EndoE-GH20 domain substrate specificity. **a**, **b** Superposition of the X-ray crystal structure of EndoE-GH20 with the X-ray crystal structure of StrH-GH20A (**a**) (PDB code: 2YL8) and StrH-GH20B (**b**) (PDB code: 2YLA). Labelled residues correspond to EndoE-GH20. **c**, **d** Ribbon representation of molecular docking calculations of EndoE-GH20 with a GlcNAc_2_Man_3_GlcNAc_2_ substrate inserting either the α(1,3) (**c**) or the α(1,6) (**d**) antenna into the active site of the enzyme. **e**, **f** Surface representation of molecular docking calculations of EndoE-GH20 with a GlcNAc_2_Man_3_GlcNAc_2_ substrate inserting either the α(1,3) (**e**) or the α(1,6) (**f**) antenna into the active site of the enzyme. The schematic representations of G0 boxed in panels **c**–**f** maintain the same orientation as the cartoon representation in the corresponding panel. The carbohydrate residues are numbered based on the sugar-binding subsites in GHs^88^. Subsites are labeled from −n to +n (where n is an integral number); −n indicates the non-reducing end and +n the reducing end of the *N*-glycan. The hydrolysis takes place between −1 and +1.

The EndoE-GH20 domain exhibits structural homology with the GH20A and GH20B domains of StrH (Fig. 9 and Supplementary Figs. 3 and 11)^54^. Each GH20 domain of StrH has a slightly different *N*-glycan specificity according to glycan microarray binding experiments^54^. The inactive GH20B domain selectively binds to the terminal GlcNAcβ-1,2-Man of α(1,3) antenna and bisecting CT *N*-glycans. In contrast, the inactive GH20A domain binds to the terminal GlcNAcβ-(1,2)-Man of both α(1,3) and α(1,6) antennae but does not recognize bisecting CT *N*-glycans^54^. The structural comparison of the GH20 EndoE with the GH20A and GH20B domains of StrH in complex with *N*-glycan substrates indicates that the substrate-binding site is essentially preserved between these enzymes (Fig. 9). The GH20 domain of EndoE cannot accommodate bisecting CT *N*-glycans due to the presence of bulky and hydrophobic residues in loop 7 that block the access of the additional β(1,4)-GlcNAc to the binding site, as in the case of the GH20A domain of StrH (Fig. 9). In addition, molecular docking calculations of CT *N-*glycans into the binding site of the GH20 domain of EndoE strongly suggest that the enzyme can accept and hydrolyze the terminal GlcNAc of both α(1,3) and α(1,6) antennae, similarly to GH20A (Fig. 9).

## Discussion

The vast majority of characterized glycoside hydrolases are proteins with a single GH domain, which may also contain other accessory domains that do not show enzymatic activity, including CBMs^15,63^. To our knowledge, EndoE is the only known CAZyme comprising a GH18 domain and a GH20 domain. The study of the molecular mechanism of substrate recognition of the individual domains of multi-modular glycoside hydrolases such as EndoE and how these domains can work synergistically is of critical importance not only to understand their mode of action in the context of a particular biological function but also to the rational design of novel enzymes that can optimize the glycoengineering of glycoproteins.

The crystal structures of EndoE-GH18L and EndoE-GH20 constructs reveal that EndoE is a unique multi-modular enzyme that harbors two glycoside hydrolases belonging to different families. The N-terminal domain comprises a GH18 enzyme while the C-terminal domain is composed of a GH20 enzyme, both connected by two consecutive 3HB domains (Figs. 1 to 3 and Supplementary Fig. 4). The ab initio low resolution calculated envelopes for EndoE displayed an elongated, asymmetric protein with two differently sized lobes separated by a deep cleft (Fig. 4). The large lobe corresponds to the EndoE-GH18L structure, comprising the GH18 domain and the two 3HB domains, whereas the small lobe resembles the GH20 domain (Fig. 4). The multi-modular architecture of EndoE reveals how the interplay of individual GH domains’ function influences biological activity.

EndoE catalyzes the hydrolysis of CT *N*-glycans in glycoproteins, but the molecular mechanism for this activity has not been understood yet. EndoE was found previously to process CT oligosaccharides into (i) IgG γ-chain^18^, (ii) human lactoferrin^25^, and (iii) trastuzumab (herceptin)^26^, an antibody that targets human epidermal growth factor receptor 2 (HER2) and has been used for the treatment of breast cancer^64,65^. This ability was associated with the endo-β-*N*-acetylglucosaminidase activity provided by the GH18 domain, and in the case of the IgG γ-chain by the GH20 domain^18^. Our activity measurements revealed that neither EndoE-GH18L nor EndoE-GH20 constructs hydrolyze the CT *N*-glycan from Rituximab, mainly composed of CT *N*-glycans with two terminal Gal in the α(1,3) and α(1,6) antennae (G2), G1, and G0. The activity of EndoE against Rituximab is restored when a mixture of both EndoE-GH18L and EndoE-GH20 constructs were incubated with the monoclonal antibody (Fig. 5; Supplementary Fig. 6). However, neither the full-length enzyme nor the combination of both domains was able to fully hydrolyze the *N*-glycan from Rituximab, suggesting that some glycoforms (G2 and some G1) are not susceptible to EndoE activity. We found that the GH20 domain is an exo-β-1,2-*N*-acetylglucosaminidase with the ability to hydrolyze the non-reducing terminal GlcNAc residue from CT *N*-glycans. Our hydrolytic assays, structural studies, and docking calculations indicate that the GH20 domain can accommodate the terminal β(1,2)-GlcNAc of the α(1,3) antenna or α(1,6) antenna of CT *N*-glycans into the binding site (Fig. 5; Fig. 9; Supplementary Fig. 11). This suggests that the GH20 domain of EndoE could hydrolyze both terminal GlcNAc to generate a GlcNAc_2_Man_3_ glycoform (Figs. 5 to 7; Supplementary Fig. 5). This enzymatic activity is necessary to reduce the size of the CT *N*-glycan. Supporting this notion, docking calculations clearly show that the active site of the GH18 domain can accommodate a CT *N*-glycan with a short α(1,3) antenna composed just of Man (-5) (Fig. 8 and Supplementary Fig. 9). However, loop 1 restricts the access of the additional GlcNAc (-6) and Gal (-7) of this antenna (Supplementary Fig. 9). However, the β(1,2)-linkage of α(1,6) antenna of CT *N*-glycans orients the GlcNAc (-4) away from the binding site of the enzyme suggesting that the GH18 domain of EndoE can accept CT *N*-glycans with longer α(1,6) antennae (Supplementary Fig. 9). Subsequently, the GH18 domain can recognize and cleave the β(1,4) linkage within the di-*N*-acetylchitobiose comprised of the CT core. This mechanism of hydrolysis by the EndoE GH18 domain requires the activity of the EndoE-GH20 domain and/or other enzymes that reduce the size of the α(1,3) antenna into a shorter glycoform.

**Fig. 10:**
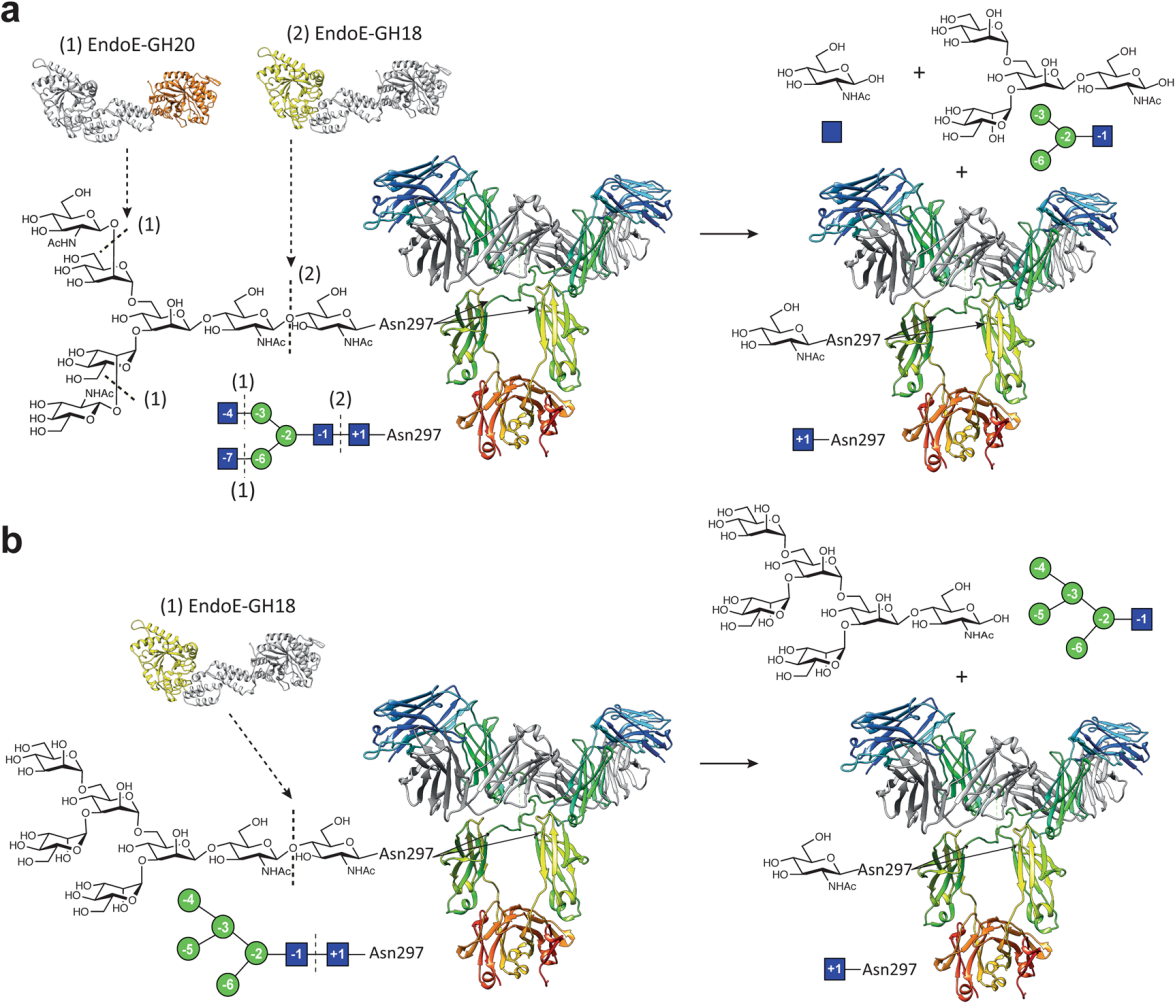
EndoE model of action. **a** Schematic representation of CT *N*-glycan processing by EndoE. In a first step, EndoE-GH20 cleaves the non-reducing terminal GlcNAc residues from the CT *N*-glycan substrate, generating smaller glycoforms that can be processed by EndoE-GH18. In a later stage, EndoE-GH18 cleaves the chitobiose core of the *N*-linked glycan, releasing the glycan from the protein substrate. **b** Schematic representation of HM-type *N*-glycan processing by EndoE. As EndoE-GH20 has no activity on mannose, EndoE-GH18 is the only domain acting on HM-type *N*-glycan substrates, directly cleaving the chitobiose core of the *N*-linked glycan and releasing the glycan from the protein substrate.

EndoE has also the ability to process shorter versions of HM-type *N*-glycans on (i) RNAse B^18^ and (ii) two recombinant versions of trastuzumab expressed in *Pichia pastoris*^26^. Specifically, the single HM-type *N*-glycan on RNAse B occurs in several different forms with varying numbers of Man residues, resulting in five RNAse B glycoforms (Man_5_–Man_9_). EndoE hydrolysis shifts the size of the smaller glycoforms of RNAse B, Man_5_, and Man_6_, into a single peak^18^. In addition, SDS-PAGE analysis clearly shows that both EndoE and EndoE-GH18L, but not EndoE-GH20, cleave HM-type *N*-glycans on RNAse B (Supplementary Fig. 5). Moreover, our LC-MS analysis for EndoE-GH18L with RNAse B indicates that this domain hydrolyzes the Man_5_ glycoform and, at a slower rate, the Man_6_ glycoform. EndoE-GH18L appears to be inactive on RNAse B bearing larger HM-type *N*-glycans containing more than two Man residues in the α(1,3) antenna. In that sense, our crystal structure of the EndoE-GH18L-Man_5_ complex unveils the molecular mechanism of HM-type *N*-glycan substrate recognition and specificity of EndoE. Supporting this notion, the crystal structure of the EndoE-GH18L-Man_5_ complex and docking calculations of EndoE-GH18L with Man_6_, Man_7_, and Man_9_ show that Man_6_ can bind to the active site of the GH18 domain because the additional mannose (Man (-7)) of the α(1,3) antenna can interact with loop 1 of the enzyme. However, additional mannose residues in this antenna as occur in the Man_7_, Man_8_, and Man_9_ glycoforms, make clashes with this same loop (Supplementary Fig. 9). Furthermore, additional mannose residues of the α(1,6) antenna of Man_9_ cannot be accommodated in the active site of the GH20 domain due to steric hindrance. The structural superposition with other ENGases capable of processing large HM-type glycans such as EndoS2 and EndoBT-3987 shows that the equivalent loops leave enough space for the long α(1,3) antenna and the branched α(1,6) antenna of the Man_9_ glycan, either because these loops are very short or because they adopt a conformation in which they do not obstruct the groove Fig. 8e, f. Therefore, the ability of EndoE to process small HM-type *N*-glycans on glycoproteins is exclusively associated with the endo-β-*N*-acetylglucosaminidase activity of the GH18 domain. The conformation that adopts the core, Man_3_GlcNAc, in the GH18 domain of EndoE is very similar to that found for the same core *N*-glycan in the EndoS2-HM-type complex (PDB code 6MDV; r.m.s.d. 2.7 Å), while this glycan shows a completely different conformation in the active site of EndoBT-3987 (PDB code 6T8L, r.m.s.d. 7.3)^60^, suggesting that the GH18 domains of EndoE and EndoS2 could share a common mechanism for *N*-glycan recognition. In summary, our experimental data support a model in which the GH20 and GH18 domains of EndoE tightly cooperate to process CT *N*-glycans on IgG antibodies (Fig. 10). Since the reaction product of the GH20 domain is itself the substrate of the GH18 domain, the overall structure of EndoE and the precise location of both GH domains linked by the 3HB-1 and 3HB-2 domains, might facilitate the access to the substrate into the active site in this second reaction, favoring the concerted action of the GH18 and GH20 domains.

The GH18 domain of EndoE has striking structural homology to EndoS and Endo-CoM, enzymes that are active exclusively on biantennary CT *N*-glycans (Supplementary Fig. 10). Binding to substrate *N*-glycans is predominantly driven by the glycoside hydrolase domain loops 1, 6, and 7 that contact the *N*-glycan pentasaccharide core and α(1,3) antenna, a mechanism that is also conserved in EndoS2. EndoS2 is structurally distinct from EndoS in glycoside hydrolase loops 3 and 4, which create additional space and form specific contacts with the α(1,6) antenna. This allows EndoS2 to bind HM-type substrates, which bear an extra antenna compared to complex biantennary substrates^16,61^. The GH18 domain of EndoE recognizes a broad range of *N*-glycans with respect to their α(1,6) antennae, including HM-type and CT *N*-glycans, including terminal Gal or GlcNAc residues (Figs. 5 and 8; Supplementary Figs. 9 and 10). In contrast, the recognition of the α(1,3) antenna is restricted to the mannose residue of the Man_3_ core. In that context, glycoform (**11**) found after the treatment of Rituximab with EndoE might represent a G1 α(1,3) antenna of a CT *N*-glycan.

By combining two GH domains with distinct glycan specificities—a GH18 domain that accommodates branched glycans with relatively short antennae and a GH20 domain that can trim certain branched glycans to lengths that are amenable to the GH18 to which it is linked—EndoE appears to have evolved successfully to cleave CT *N*-glycans in glycoproteins. It has recently been shown that *E. faecalis* expresses another ENGase, *Ef*Endo18A, a homolog of EndoE and regulated together with EndoE by the Carbon Catabolite Protein A (CcpA) transcriptional regulator, which is primarily responsible for deglycosylating HM-type glycoproteins under nutrient-limiting conditions^27^. With glycan metabolism largely being performed by *Ef*Endo18A in this manner, *E. faecalis* was perhaps free to evolve EndoE towards a multi-modal/multi-specific enzyme for deglycosylating CT glycoproteins. The most prominent examples of such are human IgG antibodies, and the ability of EndoE to deglycosylate host antibodies and render them incapable of mediated effector functions, may facilitate *E. faecalis* to evade the human immune system. Indeed, our data and those in numerous other studies^18,26,27^ have shown that EndoE can deglycosylate many glycoforms of CT *N*-glycans on IgG antibodies. The structural arrangement of the *N*-glycan binding site also allows EndoE to recognize and process small glycoforms of HM-type *N*-glycans not only on IgGs but also on other glycoproteins, being a source for nutrient acquisition, which suggests that EndoE could play a dual role in immune evasion and glycan metabolism depending on the environmental conditions encountered by *E. faecalis*.

Considering the restriction of the active site of EndoE to recognize the mannose residue of the Man_3_ core, to process both CT and HM-type *N*-glycans, this could represent a novel mechanism by which a glycoside hydrolase expands specificities. EndoE also represents a novel paradigm for how bacteria use secreted ENGases to increase survival. Moreover, these studies will allow glycoprotein engineers to create customized glycoproteins for use as biological reagents. This is especially important for rationalizing chemoenzymatic synthesis strategies for engineering immunotherapeutic human IgG antibodies, which rely on specific chemistries of *N*-glycans linked to a conserved asparagine residue in the Fc domain to induce certain biological effects.

## Methods

### Cloning, expression, and purification of EndoE, EndoE-GH18L, EndoE-GH20, and inactive EndoE mutants

The pGEXndoE (Addgene plasmid # 47714) vector encoding the EndoE gene from *E. faecalis* was kindly donated by Mattias Collin^18^. We introduced a TEV site (ENLYFQG) and a linker (GSG) sequence between the GST-tag and the residue 56 (UniProt code Q6U890) of EndoE using the FastCloning method^66^ (pGEXndoE-TEV; see Supplementary Table 3 for all primers and constructs). pGEXndoE-TEV vector were used as a template to generate the constructs EndoE-GH18 (56–370), EndoE-GH18L (56–486), EndoE-GH20 (487–837), EndoE-LGH20 (371–837) by the FastCloning method (Supplementary Table 3)^66^. The inactive constructs EndoE_E186Q_, EndoE_E662Q_, EndoE-GH18_E186Q_ and EndoE-GH20_E662Q_ were generated by QuikChange site-directed mutagenesis (Figs. 5 and 6; Supplementary Table 3)^67^. The full-length, truncated, and inactive constructs were expressed and purified as described below. *Escherichia coli* BL21(DE3) cells transformed with the corresponding plasmid were grown in LB medium supplemented with 100 μg mL^−1^ ampicillin at 37 °C. When the culture reached an OD_600_ value of 0.6–0.8, expression was induced by adding 1 mM IPTG. After ca. 16 h at 18 °C, the cells were harvested by centrifugation at 5000 × *g* for 20 min at 4 °C and resuspended in buffer A (100 mL of 50 mM Tris-HCl, pH 7.5, 500 mM NaCl) containing protease inhibitors (Thermo Scientific™, A32955) and 3 µL of Benzonase (Merck, 71205; solution A). Cells were disrupted by sonication (30 cycles of 10 s pulses with 60 s cooling intervals between the pulses, and 60% of amplitude) at 4 °C, and the suspension was centrifuged at 20,000 × g for 40 min at 4 °C. The supernatant was filtered with 0.2 μm filters and then applied into a GSTrap FF column (5 mL, GE Healthcare) equilibrated with buffer A. The elution was performed with a linear gradient from 0 to 100% of buffer B (50 mM Tris-HCl pH 7.5, 100 mM NaCl, 10 mM reduced glutathione) at 1 mL min^−1^. The protein was dialyzed against a dialysis buffer (50 mM Tris-HCl pH 8, 150 mM NaCl, 1 mM DTT, 0.5 mM EDTA), with TEV protease (1:20 ratio), overnight at 18 °C. The completeness of the enzymatic digestion reaction was confirmed by SDS-PAGE and the protein was loaded into a GSTrap FF column (5 mL, GE Healthcare) equilibrated with buffer A. Flow-through was loaded into a HisTrap HP (5 mL, GE Healthcare). The elution was performed with a linear gradient from 0 % to 100 % of buffer C (250 mM Tris-HCl pH 7.5, 250 mM NaCl, 500 mM imidazole) at 1 mL min^−1^. The flow-through was concentrated in an Amicon Ultra-15 centrifugal filter unit (Millipore) with a molecular cutoff of 30 kDa and further purified by size-exclusion chromatography using a Superdex 200 26/600 column (350 mL; GE Healthcare) equilibrated in 20 mM Tris-HCl pH 7.5, 150 mM NaCl. In the case of EndoE-GH18L and EndoE-GH20 used for crystallization experiments, the size-exclusion chromatography step was performed in a column equilibrated in 20 mM Tris-HCl pH 7.5. The eluted EndoE-GH18L and EndoE-GH20 were concentrated at 15 and 20 mg mL^−1^, respectively. The EndoE, EndoE-GH18, EndoE-GH18L, EndoE-GH20, EndoE-LGH20 (371–837), EndoE_E186Q_, EndoE_E662Q_, EndoE-GH18_E186Q_, and EndoE-GH20_E662Q_ used for hydrolytic experiments were flash-frozen and stored at −80 °C until ready for use.

### EndoE-GH18L, EndoE-GH18L-Man_5_, and EndoE-GH20 crystallization and data collection

EndoE-GH18L was crystallized by mixing 0.25 µL of a protein solution at 15 mg mL^−1^ in 20 mM Tris-HCl pH 7.5 with 0.25 µL of PEG/Ion HT screening condition H8, Hampton Research (20 mM zinc chloride, 20% *w/v* PEG 3350). Crystals were transferred to a cryo-protectant solution containing 30% glycerol and frozen under liquid nitrogen. Complete X-ray diffraction datasets were collected at the beamline X06DA (PXIII) of the Swiss Light Source (SLS, Villigen, Switzerland). EndoE-GH18L crystallized in the *P* 6 5 space group with one molecule in the asymmetric unit and diffracted to a maximum resolution of 1.7 Å (Supplementary Table 1). The EndoE-GH18L-Man_5_ complex was crystallized by mixing 0.25 µL of a protein solution at 15 mg mL^−1^ in 20 mM Tris-HCl pH 7.5 and 2.5 mM Man_5_GlcNAc with 0.25 µL of PACT premier HT-96 / FX-96 screening condition F5, Molecular Dimension (100 mM Bis-Tris propane pH 6.5, 200 mM sodium nitrate, 20% *w/v* PEG 3350). Crystals were transferred to a cryo-protectant solution containing 30% glycerol and frozen under liquid nitrogen. Complete X-ray diffraction datasets were collected at the beamline BL13-XALOC (ALBA, Cerdanyola del Valles, Spain). EndoE-GH18L-Man_5_ crystallized in the *C* 1 2 1 space group with two molecules in the asymmetric unit and diffracted to a maximum resolution of 2.6 Å (Supplementary Table 1). EndoE-GH20 was crystallized by mixing 0.25 µL of a protein solution at 20 mg mL^−1^ in 20 mM Tris-HCl pH 7.5 and 2.5 mM CT *N*-glycan (NeuAc_2_Gal_2_GlcNAc_2_Man_3_GlcNAc(Fuc)GlcNAc) with 0.25 µL of Morpheus screening condition A12, Molecular Dimensions (100 mM bicine/Trizma base pH 8.5, 30 mM magnesium chloride hexahydrate, 30 mM calcium chloride dihydrate, 12.5% *w/v* PEG 1000, 12.5% PEG *w/v* 3350, 12.5% *v/v* MPD). A single crystal grew after 15 days. Complete X-ray diffraction datasets were collected at the beamline I24 (Diamond Light Source, Oxfordshire, UK). GH20 crystallized in the *P* 1 2 1 1 space group with one molecule in the asymmetric unit and diffracted to a maximum resolution of 1.4 Å (Supplementary Table 1). All datasets were integrated and scaled with XDS following standard procedures^68^.

### EndoE-GH18L, EndoE-GH18L-Man_5_, and EndoE-GH20 structures determination and refinement

The structure determination of EndoE-GH18L was carried out using MrBump^69^ and MolRep^70^, using the coordinates of the endo-β-*N*-acetylglucosaminidase EndoS2 from *S. pyogenes* (PDB code 6MDV), as a search model^61^. The structure determination of EndoE-GH18L-Man_5_ and EndoE-GH20 was performed by molecular replacement methods implemented in Phaser^71^ and the PHENIX suite^72^, using the coordinates of EndoE-GH18L (PDB code 7PUJ) and that of the β-*N*-acetyl-hexosaminidase StrH from *Streptococcus pneumoniae* R6 (PDB code 3RPM), as search templates respectively. The model rebuilding was carried out with Buccaneer^73^ and the *CCP4* suite^74^. The final manual building was performed with Coot^75^ and refinement with phenix.refine^76^. The structure was validated by MolProbity^77^. The structure of the Man_5_ product was validated by Privateer^78^. Data collection and refinement statistics are presented in Supplementary Table 1. The atomic coordinates and structure factors were deposited in the Protein Data Bank, accession codes 7PUJ (EndoE-GH18L), 7PUK (EndoE-GH20), 7PUL (EndoE-GH18L-Man_5_). Molecular graphics and structural analyses were performed with the UCSF Chimera package^79^.

### SEC-SAXS experiments

Small-Angle X-ray Scattering coupled with Size Exclusion Chromatography (SEC-SAXS) data for recombinant purified EndoE (56–837) in 50 mM Tris-HCl pH 7.5, 100 mM NaCl, 2% *v/v* glycerol were collected on the B21 beamline of the Diamond Light Source, UK. Data were collected using a Pilatus2M detector (Dectris, CH) at a sample-detector distance of 3914 mm and a wavelength of λ = 1 Å. The range of momentum transfer of 0.1 < s < 5 nm−1 was covered (s = 4πsinθ/λ, where θ is the scattering angle). 50 µL of a protein sample at 10 mg mL^−1^ were injected into a Shodex KW403-4F column and eluted at a flow rate of 150 μL min^−1^. Data were processed and merged using standard procedures by the program package ScÅter^80^ and PRIMUS^81^. The maximum dimensions (*D*_*max*_), the interatomic distance distribution functions (*P(r)*), and the radii of gyration (*Rg*) were computed using GNOM^82^. The molecular mass was determined using ScÅter^80^. The ab initio SAXS envelope of full EndoE was calculated using GASBOR^83^. The results and statistics are summarized in Supplementary Table 2. The molecular weight from the SAXS data was calculated using SAXSMoW^84^. The EndoE was obtained by fitting the crystal structures of EndoE-GH18L and EndoE-GH20 in the ab initio envelope of EndoE and the missing loop between crystal structures (483–488) was built using the Robetta Web Server^85^.

### EndoE activity assays

For the enzymatic activity assays analyzed by SDS-PAGE, 100 µL reactions were setup containing 1.6 μM Rituximab or 4 μM RNAse B mixed with 0.32 μM of either the purified full-length enzyme, EndoE-GH20 in PBS and incubated at 37 °C for 2 h. In the case of RNAse B, mixtures of 4 μM RNAse A with the corresponding EndoE constructs were prepared as a control.

For the LC-MS enzymatic activity assays, 20 µL reactions were setup containing 1 µM of RNAse B (NEB), Rituximab ± BgaA galactosidase or human transferrin (Sigma–Aldrich)± MvNA sialidase±BgaA galactosidase and 1 µM of enzyme in PBS pH 7.4. In the case where inactive and active enzymes were incubated together, equimolar amounts were added to the reaction. The reactions were allowed to proceed for 10 days at room temperature before being analyzed by LC-MS. The reactions were analyzed by LC-MS using an Agilent 1290 Infinity II LC System equipped with a 50 mm PLRP-S column from Agilent with 1000 Å pore size. The LC system is attached to an Agilent 6560 Ion Mobility (IM) quadrupole- time of flight (Q-TOF) mass spectrometer (Agilent, Santa Clara, CA). Relative amounts of the substrate and hydrolysis products were quantified after deconvolution of the raw data and identification of the corresponding peaks using BioConfirm (Agilent, Santa Clara, CA). All reactions were performed in triplicate.

For the LC-MS kinetic analysis, 30 μL reactions were setup containing 1 µM Rituximab treated with BgaA galactosidase and 500 nM of either EndoE or 500 nM each of EndoE-GH18L + EndoE-GH20. The reactions were setup and placed in the same LC-MS setup as above. The reactions were sampled approximately every 15 min for 12 hs. All reactions were performed in triplicate. The data was extracted with UNIDEC^86,87^. The data was then imported into Kintek Global Kinetic Explorer^88^ for data fitting. The experimental data and fitted models are shown in Supplementary Fig. 12.

Kinetic analysis of GH20 domain. The kinetic assays were performed with 4-nitrophenol (4NP) linked substrates, 4NP-galactose, 4NP-β-*N*-acetylglucosamine, and 4NP-mannose, purchased from Sigma–Aldrich. All assays were performed in PCTP buffer pH 8 (sodium propionate, sodium cacodylate trihydrate, and Bis-Tris propane) (Molecular Dimensions). To determine the substrate specificity of the GH20 domain, 100 µL reactions were setup containing 1 mM of either 4NP-galactose, 4NP-β-*N*-acetylglucosamine and 4NP-mannose, and 50 nM of EndoE. The kinetic assays were performed with 12–0.09 mM substrate and 5 nM of either EndoE, EndoE-GH20, or EndoE-GH20_E662Q_. The reactions were allowed to proceed for 45 min. The absorbance for all experiments was read at 420 nm using a Cytation 5 plate reader in 96-well curved bottom plates, with no enzyme controls subtracted. All reactions were performed in triplicate. Kinetic parameters *k*_cat_ and *K*_m_ were determined by plotting V_0_ versus substrate concentration and fit to Michelis-Menten curve using Graphpad Prism.

### Molecular docking calculations

The HM-type (Man_6_, Man_7_, Man_9_), CT (G2, G1(α1-3), G1(α1-6), G0), and Hy-type products used in docking calculations were modeled using GLYCAM-Web website (Complex Carbohydrate Research Center, University of Georgia; http://www.glycam.com). Ligand docking was performed using AutoDock Vina employing standard parameters^62^.

### Reporting summary

Further information on research design is available in the Nature Research Reporting Summary linked to this article.

## Supplementary information

Supplementary Information

Reporting Summary

## Data Availability

The atomic coordinates and structure factors have been deposited with the Protein Data Bank (PDB), accession codes 7PUJ (EndoE-GH18L), 7PUK (EndoE-GH18L-Man_5_), and 7PUL (EndoE-GH20). Previously published PDB structures used in this study are available under the accession codes: 6KPO, 4NUY, 6E58, 4NUZ, 2OZN, 1YWM, 2YL8, 1YHT, 6MDV, 6T8L, 5BXR, 3RPM, 2YLA. All other data are available from the corresponding authors upon reasonable request. Source data are provided with this paper.

## Source data

Source Data
