# Supplementary material for: Mechanism of cooperative N-glycan processing by the multi-modular endoglycosidase EndoE
Source: Nat Commun. 2022 Mar 3;13:1137. doi: 10.1038/s41467-022-28722-w (PMC8894350; doi:10.1038/s41467-022-28722-w)
Supplement: Supplementary file 1 — Supplementary Information [file 41467_2022_28722_MOESM1_ESM.pdf]

SUPPLEMENTARY INFORMATION

Mechanism of cooperative *N*-glycan processing by the multi-modular endoglycosidase EndoE

Mikel García-Alija,[1,2,#] Jonathan J. Du,[3,#] Izaskun Ordóñez,[2] Asier Diz-Vallenilla,[2] Alicia Moraleda-Montoya,[1,2] Nazneen Sultana,[3] Chau G. Huynh,[3] Chao Li,[4] Thomas Connor Donahue,[4] Lai-Xi Wang,[4] Beatriz Trastoy,[1,2,*] Eric J. Sundberg,[3,*] and Marcelo E. Guerin.[1,2,5,*]

[1]Structural Glycobiology Laboratory, Biocruces Bizkaia Health Research Institute, Cruces University Hospital, 48903 Barakaldo, Bizkaia, Spain
[2]Structural Glycobiology Laboratory, Center for Cooperative Research in Biosciences (CIC bioGUNE), Basque Research and Technology Alliance (BRTA), Bizkaia Technology Park, Building 801A, 48160 Derio, Spain
[3]Department of Biochemistry, Emory University School of Medicine, Atlanta, GA 30322, USA
[4]Department of Chemistry and Biochemistry, University of Maryland, College Park, MD 20742, USA
[5] Ikerbasque, Basque Foundation for Science, 48009 Bilbao, Spain

[#]These authors contributed equally

*To whom correspondence should be addressed: Beatriz Trastoy, Structural Glycobiology Laboratory, IIS-BioCruces Bizkaia Cruces Plaza, 48903 Barakaldo, Bizkaia, Spain, beatriz.trastoy@gmail.com; Eric J. Sundberg, Department of Biochemistry, Emory University School of Medicine, Atlanta, GA 30322, USA, eric.sundberg@emory.edu; Marcelo E. Guerin, Structural Glycobiology Laboratory, IIS-BioCruces Bizkaia Cruces Plaza, 48903 Barakaldo, Bizkaia, Spain, mrcguerin@gmail.com.

Short title: *Cooperative processing of N-glycans by a multi-modular endoglycosidase*

Keywords: endo-β-*N*-acetylglucosaminidases, glycoside hydrolases, glycoprotein bioengineering, antibody glycoengineering, enzyme specificity, carbohydrate active enzymes, bacterial pathogenesis.

Table of Contents

1. Supplementary Tables

Supplementary Table 1: X-ray data collection and refinement statistics.

Supplementary Table 2: SAXS data and refinement parameters.

Supplementary Table 3: Summary of constructs.

Supplementary Table 4: Theoretical mass and experimentally determined mass for each annotated peak in the LC-Ms experiments

2. Supplementary Figures

Supplementary Figure 1: Recombinant production of EndoE constructs.

Supplementary Figure 2: Electron density maps of the refined EndoE X-ray crystal structures.

Supplementary Figure 3: The catalytic mechanisms of GH18 and GH20.

Supplementary Figure 4: Structural homologues of EndoE.

Supplementary Figure 5: The overall structure of the 'linker region' of EndoE.

Supplementary Figure 6: Molecular basis of EndoE GH18 and GH20 domains substrate specificity.

Supplementary Figure 7: Molecular basis of EndoE GH18 and GH20 domains substrate specificity visualized by LC-MS. Rituximab and

Supplementary Figure 8: Molecular basis of EndoE GH18 and GH20 domains substrate specificity visualized by LC-MS. Transferrin.

Supplementary Figure 9: Structural basis of GH18 substrate specificity.

Supplementary Figure 10: Sequence alignment of GH18 with homologues.

Supplementary Figure 11: Sequence alignment of GH20 with homologues.

Supplementary Figure 12: Kinetic modeling of deglycosylation of G0/G0-Rituximab by EndoE.

1. Supplementary Tables

Supplementary Table 1. X-ray data collection and refinement statistics.

	EndoE-GH18L	EndoE-GH18LMan5	EndoE-GH20
PDB code	7PUJ	7PUK	7PUL
Beamline	X06DA-PXIII (SLS)	BL13-XALOC (ALBA)	I24 (DLS)
Wavelength (Å)	0.9792	0.979	0.9686
Resolution range (Å)	38.82 - 1.752 (1.815 - 1.752)	45.73 – 2.69 (2.786 – 2.69)	44.08 - 1.4 (1.45 - 1.4)
Space group	P 65	C 1 2 1	P 1 21 1
Unit cell (Å)	112.15 112.15 64.62 90 90 120	222.03 54.55 83.92 90 110.36 90	39.2985 57.0459 69.5253 90 92.969 90
Total reflections	890490 (59382)	115391 (10126)	190656 (18241)
Unique reflections	46562 (4569)	26261 (2438)	60346 (6021)
Multiplicity	19.1 (13.0)	4.4 (4.2)	3.2 (3.0)
Completeness (%)	99.89 (99.05)	98.75 (92.80)	99.69 (99.52)
Mean I/sigma(I)	34.02 (2.73)	10.16 (1.90)	11.92 (2.12)
Wilson B-factor ($Å^2$)	25.47	62.82	12.65
R-merge	0.06049 (0.9085)	0.1232 (0.8459)	0.07721 (0.2442)
R-meas	0.06211 (0.9456)	0.1415 (0.9717)	0.09335 (0.2952)
CC1/2	1 (0.839)	0.992 (0.678)	0.991 (0.894)
CC*	1 (0.955)	0.998 (0.899)	0.998 (0.971)
Reflections used in refinement	46559 (4568)	26247 (2435)	60320 (6015)
Reflections used for R-free	2328 (229)	1313 (121)	3047 (307)
R-work	0.1862 (0.2594)	0.2284 (0.4083)	0.1578 (0.1980)
R-free	0.2203 (0.2793)	0.2618 (0.4745)	0.1829 (0.2424)
CC(work)	0.960 (0.860)	0.937 (0.654)	0.967 (0.917)
CC(free)	0.947 (0.849)	0.942 (0.333)	0.955 (0.865)
Number of non-hydrogen atoms	3575	6787	3118
macromolecules	3343	6647	2805
ligands	6	140	4
Protein residues	422	847	352
RMS(bonds) (Å)	0.006	0.002	0.008
RMS(angles) (º)	0.79	0.51	0.95
Ramachandran favored (%)	97.62	97.27	97.41
Ramachandran allowed (%)	2.38	2.73	2.59
Ramachandran outliers (%)	0.00	0.00	0.00
Rotamer outliers (%)	0.00	0.58	0.34
Clashscore	1.37	1.37	0.00
Average B-factor ($Å^2$)	30.46	62.62	16.16
macromolecules	30.22	62.59	15.34
ligands	28.49	64.10	16.87
solvent	34.16		23.59

Statistics for the highest-resolution shell are shown in parentheses

Supplementary Table 2. SAXS data collection and refinement parameters.

Data collection parameters	EndoE
**Instrument**	B21 (DLS)
**Wavelength (Å)**	0.99
**S range ($Å^{-1}$)**	0.002-0.420
**Exposure time (s per frame)**	0.56
**Concentration range (mg/ml)**	10
**Temperature (K)**	298
**Structure parameters**	
***I*(0) (a.u.)[1] (from *P(r)*)**	0.1
***Rg* (Å) (from *P(r)*)**	46.5
***I*(0) (a.u.)[1] (from Guinier)**	0.1
***Rg* (Å) (from Guinier)**	46.4
**D_{max} (Å)**	140
**Porod volume estimate ($Å^3$)**	168287
**Dry volume calculated from sequence ($Å^3$)[2]**	107050
**Ab initio modeling (χ^2 value)**	1.8± 0.1
**Molecular mass determination[3]**	
**Molecular mass (kDa)**	90.1
**Calculated monomeric from sequence (kDa)**	88.3
**Discrepancy (%)**	2
**Software employed**	
**Primary data reduction**	*SCÅTTER*
**Data processing**	*PRIMUS/SCÅTTER*
**Ab initio analysis**	*GASBOR*

[1] arbitrary units
[2] Dry volume determined using the server: http://biotools.nubic.northwestern.edu/proteincalc.html
[3] Molecular weight determination by SAXSMoW

Supplementary Table 3. Summary of constructs.

Description	Sequence (UniProt code Q6U890)	Template	Primes (5' to 3')
EndoE	56-837	pGEXndoE	addgene[1]
EndoE TEV site	56-837	pGEXndoE-TEV	1)TGTATTTTCAGGGCGGAAGTGGAAATGAGCCGACCCAAGAGAAACATTTTATGGTTTATTATCG<br>2)TCCGCCCTGAAAATACAGGTTTTCGGGGATCCCACGACCTTCGATCAGATCC
EndoE-GH18	56-370	pGEXndoE-TEV	1)ACACAGACTAGCTCGAGCGGCCGCATCGTG<br>2)AAAACGAATTAGCTACGCCAACACAGACTAGCTCGAGCG
EndoE-GH18L	56-486	pGEXndoE-TEV	1)AAGAGAAATAGCTCGAGCGGCCGCATCGTGAC<br>2)CGGAGGTCCAAACAGCTTTGGATAATTTAAAAGAGAAATAGCTCGA
EndoE-GH20	487-837	pGEXndoE-TEV	1)GAAGTGGACAAACACAACCTTTAAAAAGTGTCTTCTCCATTGATGCG<br>2)CCCCGAAAACCTGTATTTTCAGGGCGGAAGTGGACAAACACA
EndoE-LGH20	371-837	pGEXndoE-TEV	1)GAAGTGGACTTGAAGCACAAAATAAAGCCGCAGAAGC<br>2)CGAAAACCTGTATTTTCAGGGCGGAAGTGGACTTGAAGC
$EndoE_{E186Q}$<br>$EndoE\text{-}GH18L_{E186Q}$	56-837<br>56-486	pGEXndoE-TEV<br>EndoE-GH18L	1)GAATTGATGGGTTAGATATTGACATGCAAACTCGTCCAAGTGAAAAAGATATTG<br>2)CAATATCTTTTTCACTTGGACGAGTTTGCATGTCAATATCTAACCCATCAATTC
$EndoE_{E662Q}$<br>$EndoE\text{-}GH20_{E662Q}$	56-837<br>487-837	pGEXndoE-TEV<br>EndoE-GH20	1)GTGAAATTTTCAATTTTGGTGGCGATCAGTATGCAAATGATGTCGACACAGG<br>2)CCTGTGTCGACATCATTTGCATACTGATCGCCACCAAAATTGAAAATTTCAC

[1] pGEXndoE was a gift from Mattias Collin & Vincent Fischetti (Addgene plasmid # 47714; http://n2t.net/addgene:47714 ; RRID:Addgene_47714)

Supplementary Table 4. Theoretical mass and experimentally determined mass for each annotated peak in the LC-Ms experiments

	Glycoform	Theoretical mass (Da)	Observed mass (Da)
Rituximab	1	147074.985	147076
	2	147237.126	147233
	3	147399.264	147398
	4	147561.408	147559
	5	147723.549	147715
	6	146668.985	146670
	7	147033.985	147035
	8	147398.985	147400
	9	147763.985	147765
	10	144884.985	144886
	11	145938.985	145940
	12	146303.985	146305
	13	146871.985	146873
	14	147074.985	147076
	15	147236.985	147238
	16	147439.985	147441
	17	147601.985	147603
RnaseB	Glycoform	Theoretical mass (Da)	Observed mass (Da)
	1	13885	13884
	2	14898	14899
	3	15060	15061
	4	15222	15223
	5	15384	15384
Transferrin	Glycoform	Theoretical mass (Da)	Observed mass (Da)
	1	79282	79264
	2	79573	79556
	3	79719	79701
	4	80229	80213
	5	80375	80356
	6	78409	78391
	7	78555	78538
	8	78774	78756
	9	78920	78902
	10	77761	77744
	11	77907	77890
	12	77964	77947
	13	75571	75554
	14	75717	75700
	15	76949	76932
	16	77095	77078

2. Supplementary Figures

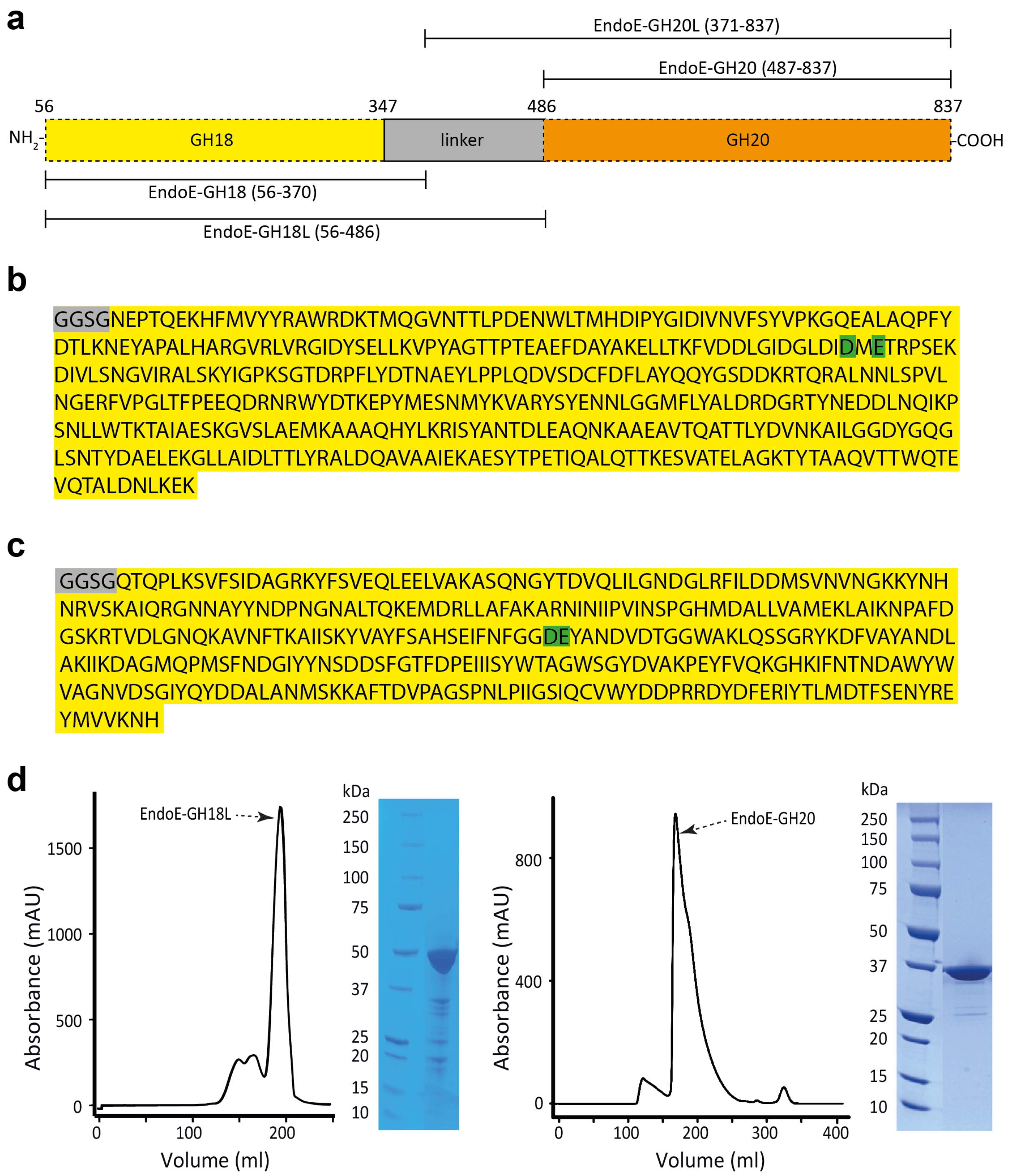

Supplementary Fig 1 | EndoE constructs. a Schematic representation of EndoE sequence and the EndoE-GH18, EndoE-GH18L, EndoE-GH20 and EndoE-GH20L constructs. The amino acid sequences of the EndoE-GH18L (**b**) and EndoE-GH20 (**c**) constructs are shown in yellow. The catalytic residues are colored in green and the GGSG spacer sequence resulting from TEV digestion is colored in gray. **d** Pre-crystallization SEC purification and SDS-PAGE analysis of EndoE-GH18L (left panel) and EndoE-GH20 (right panel). This experiment was repeated three times independently with similar results.

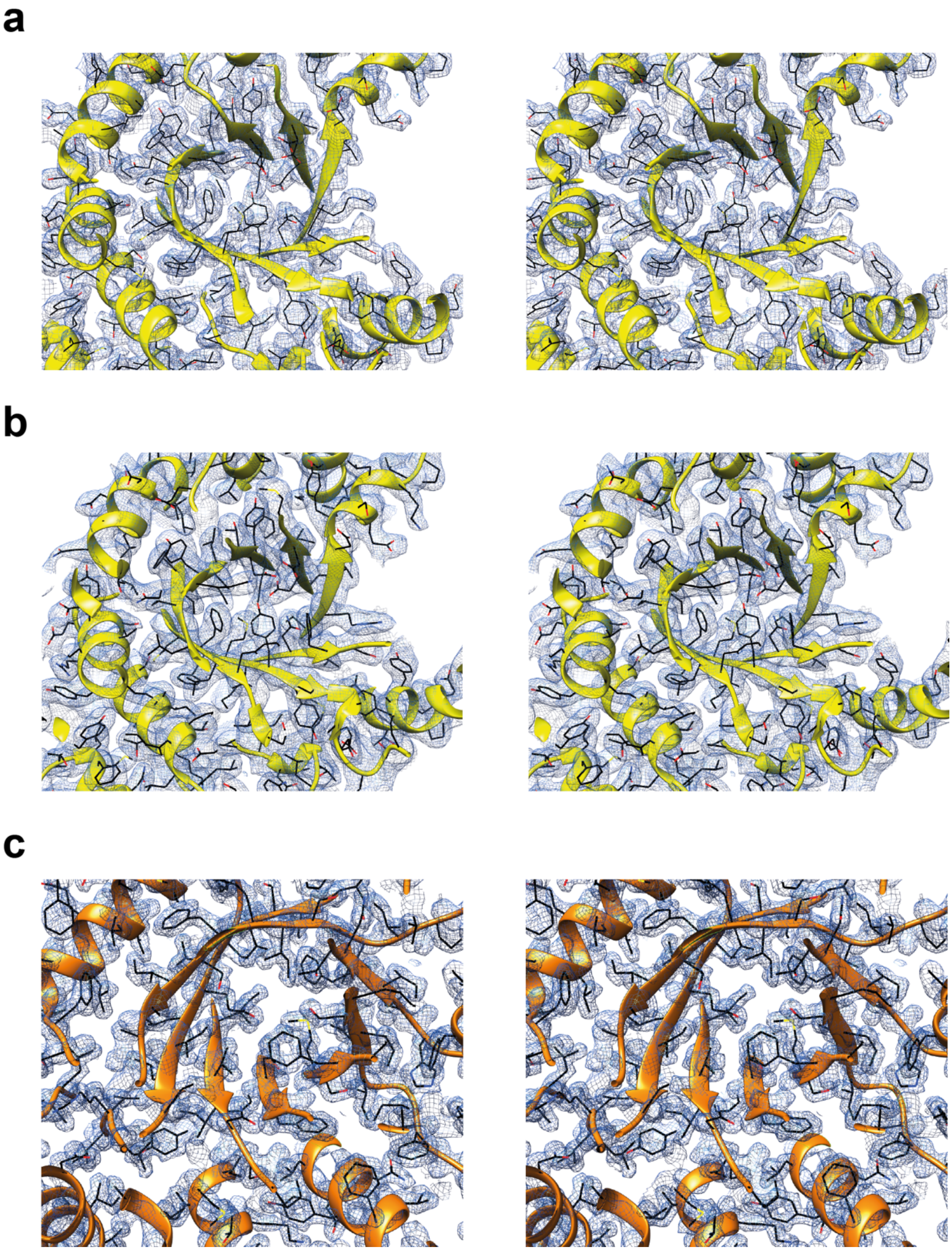

Supplementary Fig. 2 | Electron density maps of the refined structures. Final electron density maps (2mFo-DFc contoured at 1σ) corresponding to unliganded EndoE-GH18L (**a**), EndoE-GH18L in complex with $Man_5GlcNAc$ (**b**) and EndoE-GH20 (**c**). Cross-eyed stereo figures are shown.

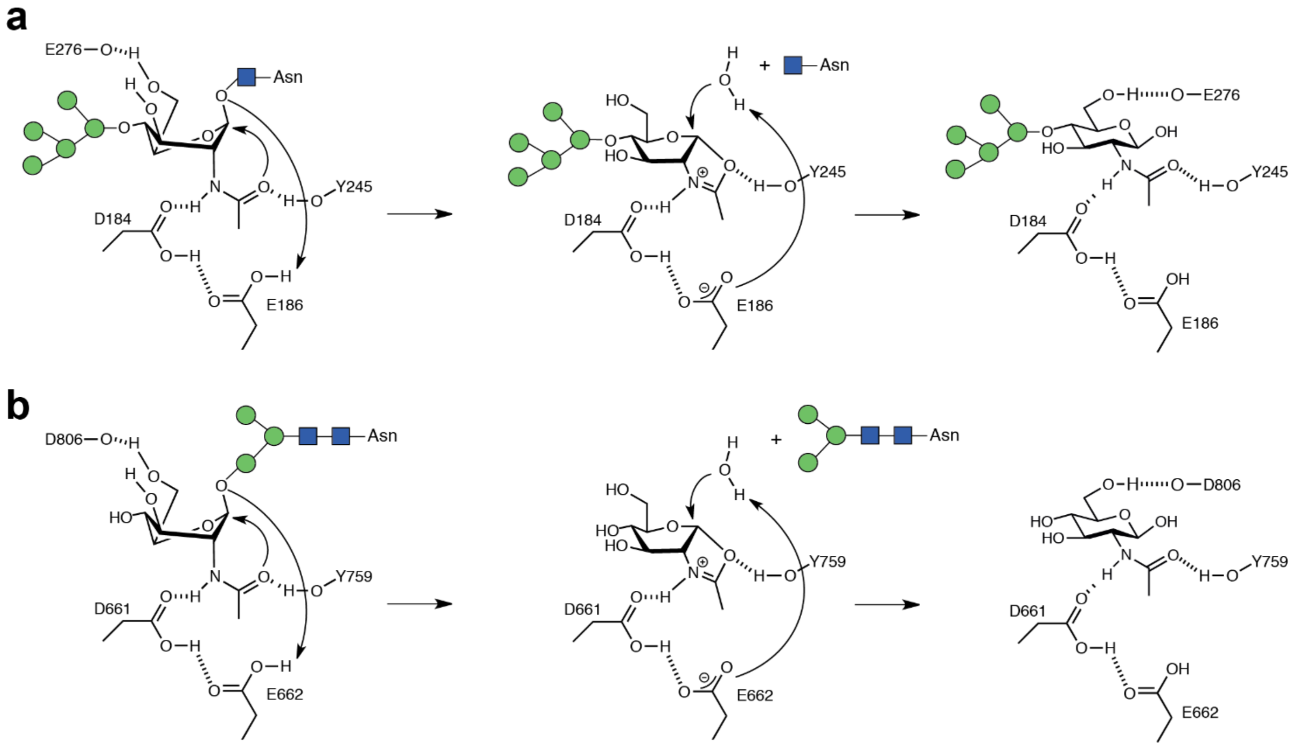

Supplementary Figure 3 | The catalytic mechanisms of EndoE-GH18 (a) and EndoE-GH20 (b). In the first step, the *N*-linked glycan substrate binds to the active site of the enzyme, which generates a distortion of GlcNAc (-1). The catalytic residues E186 or E662 act as an acid and protonate the glycosidic bond, while residues D184 or D661 interact with the nitrogen atom C2-acetamide group of GlcNAc (-1), orienting the oxygen of the C2-acetamide group into a position where it can attack the anomeric carbon of GlcNAc (-1), leading to the formation of an oxazolinium intermediate. In a second step, E186 or E662 act as a base to deprotonate a water molecule which subsequently attacks and breaks the oxazoline ring, leading to the regeneration of the hemiacetal sugar with net retention of the anomeric configuration.

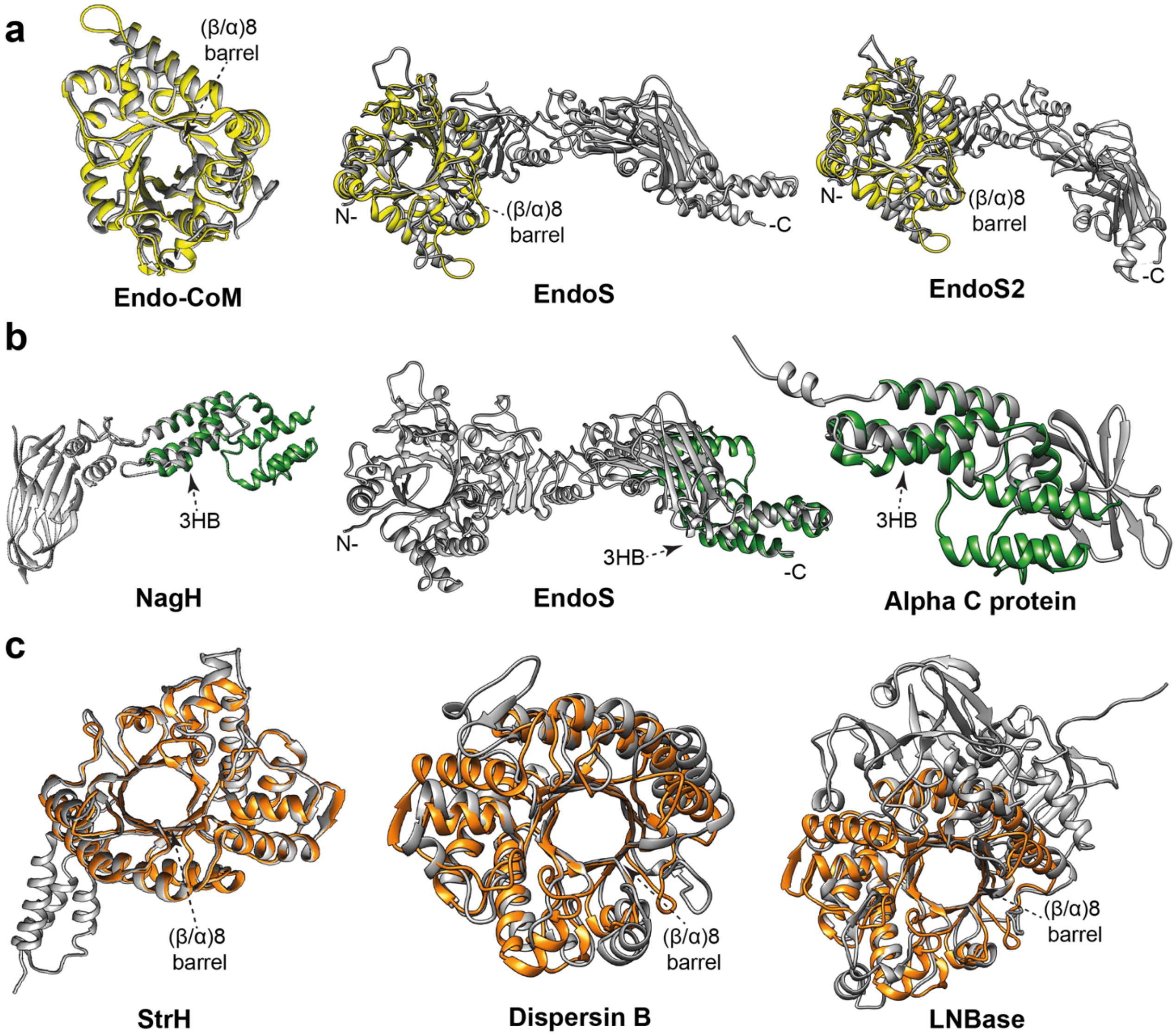

Supplementary Fig. 4 | Structural homologues of EndoE. a Superposition of the GH18 domain from EndoE (yellow) with Endo-CoM from *Cordyceps militaris* (PDB: 6KPO; left; grey), EndoS from *Streptococcus pyogenes* (PDB: 4NUY; center; grey) and EndoS2 from *Streptococcus pyogenes* (PDB: 6E58; right; grey). **b** Superposition of the linker domain from EndoE (green) with NagH from *Clostridium perfringens* (PDB: 2OZN; left; grey), EndoS from *Streptococcus pyogenes* (PDB: 4NUZ; center; grey) and alpha C protein from *Streptococcus agalactiae* (PDB: 1YWM; right; grey). **c** Superposition of the GH20 domain from EndoE (orange) with StrH from *Streptococcus pneumoniae* (PDB: 2YL8; left; grey), dispersin B from *Actinobacillus actinomycetemcomitans* (PDB: 1YHT; center; grey) and LNBase from *Bifidobacterium bifidum* (PDB: 5BXR; right; grey).

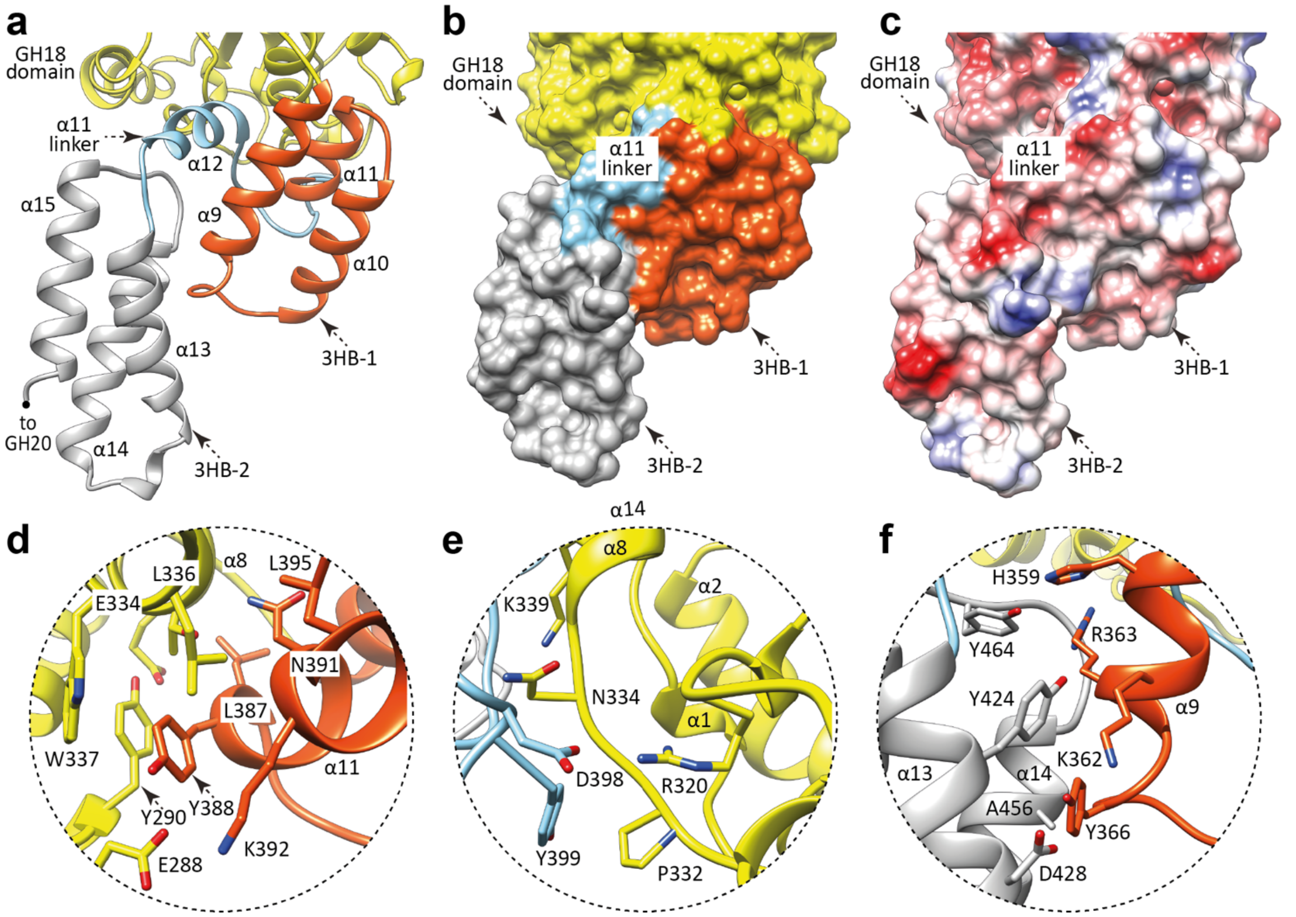

Supplementary Fig. 5 | The overall structure of the 'linker region' of EndoE. a Cartoon representation showing the general fold and secondary structure organization of 'linker region' of EndoE, including the 3HB-1 (dark orange) and 3HB-2 (light grey) domains, and the α11 linker (light blue). **b** Surface representation of 'linker region' of EndoE. **c** Electrostatic surface representations of 'linker region' of EndoE. **d** Close up view showing the interaction between the GH18 (yellow) and 3HB-1 (dark orange) as cartoon/stick representation. **e** Close up view showing the interaction between the GH18 (yellow) and the α11 linker (light blue) as cartoon/stick representation. **f** Close up view showing the interaction between the 3HB-1 (dark orange) and the 3HB-2 (light grey) as cartoon/stick representation.

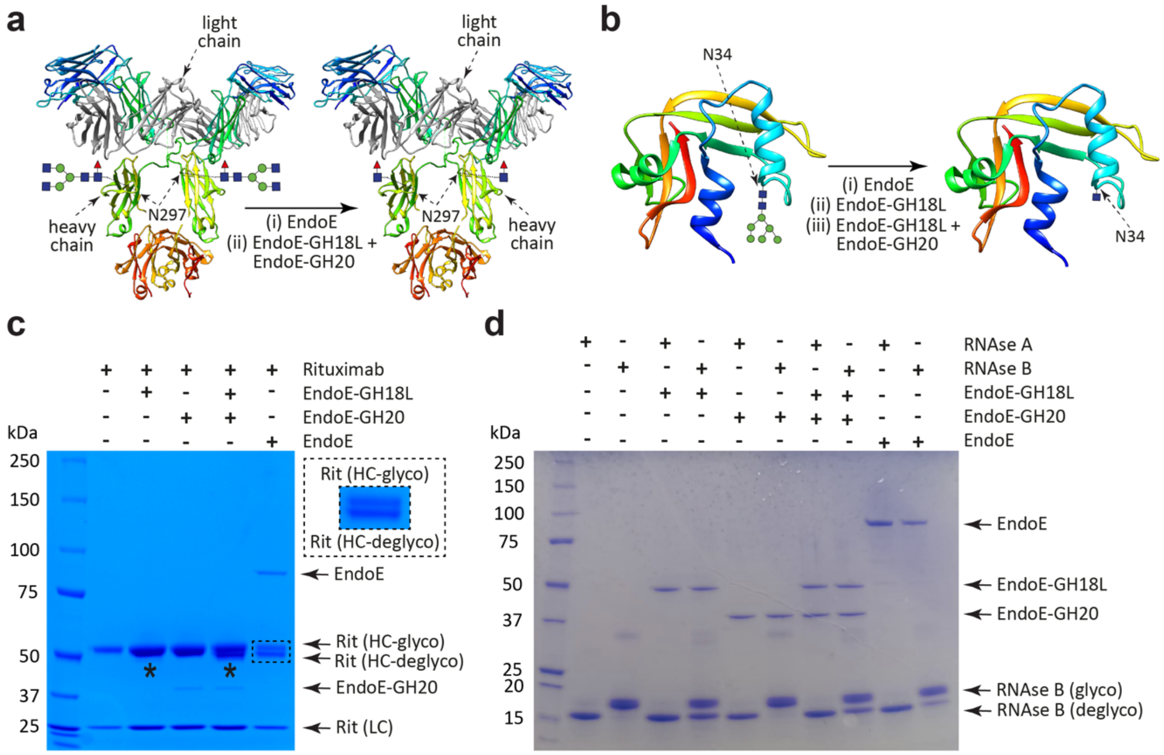

Supplementary Fig. 6 | Molecular basis of EndoE GH18 and GH20 domains substrate specificity. Enzymatic activity assays with EndoE and the individual GH18 and GH20 domains against: Rituximab (**a**) and RNAse B (**b**). **c** SDS-PAGE analysis of EndoE, EndoE-GH18L and GH20 constructs enzymatic activity against Rituximab. EndoE-GH18L is indicated with an asterisk. Light Chain and Heavy Chain of Rituximab are referred as LC and HC, respectively. Rit (HC-glyco) and Rit (HC-deglyco) refer to the glycosylated and deglycosylated forms of the Rituximab HC. The *inset panel* shows the zoom of the two distinct bands, corresponding to the HC-glyco (upper) and HC-deglyco (lower) forms of Rituximab. **d** analysis of EndoE, EndoE-GH18L and GH20 constructs enzymatic activity against RNAse A or RNAse B. This experiment was repeated two times independently with similar results.

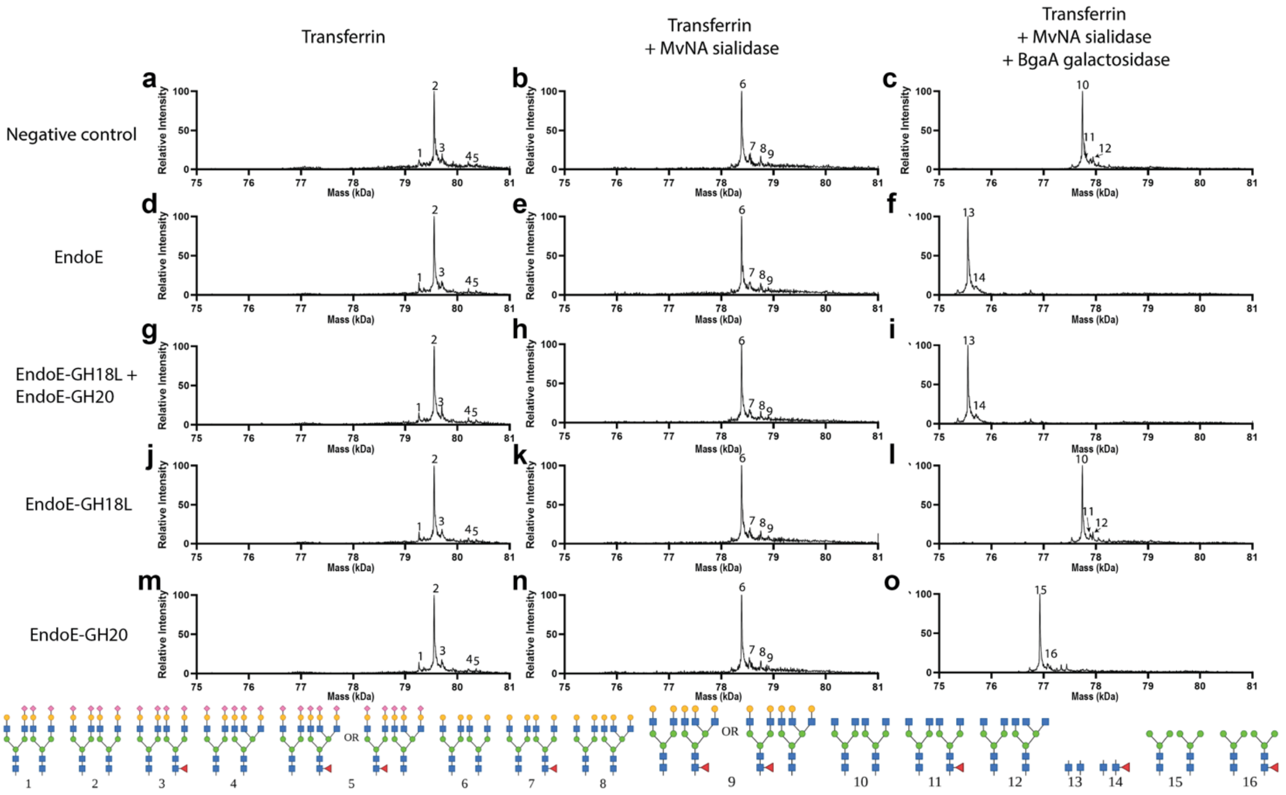

Supplementary Fig. 7 | Molecular basis of EndoE GH18 and GH20 domains substrate specificity as visualized by LC/MS. Transferrin. Processing of CT *N*-glycans on transferrin ± MvNA sialidase ± BgaA galactosidase. Mass spectrometry of transferrin ± MvNA sialidase ± BgaA galactosidase with **a b c** no enzyme **d e f** EndoE **g h i** EndoE-GH18L + EndoE-GH20 **j k l** EndoE-GH18L and **m n o** EndoE-GH20. The retention time for transferrin was 2.3 min. For mass deconvolution, the following parameters were used in the BioConfirm software; 1800-3000 m/z and 75-81 kDa. The theoretical and observed mass of each annotated peak are in Supplementary Table 4.

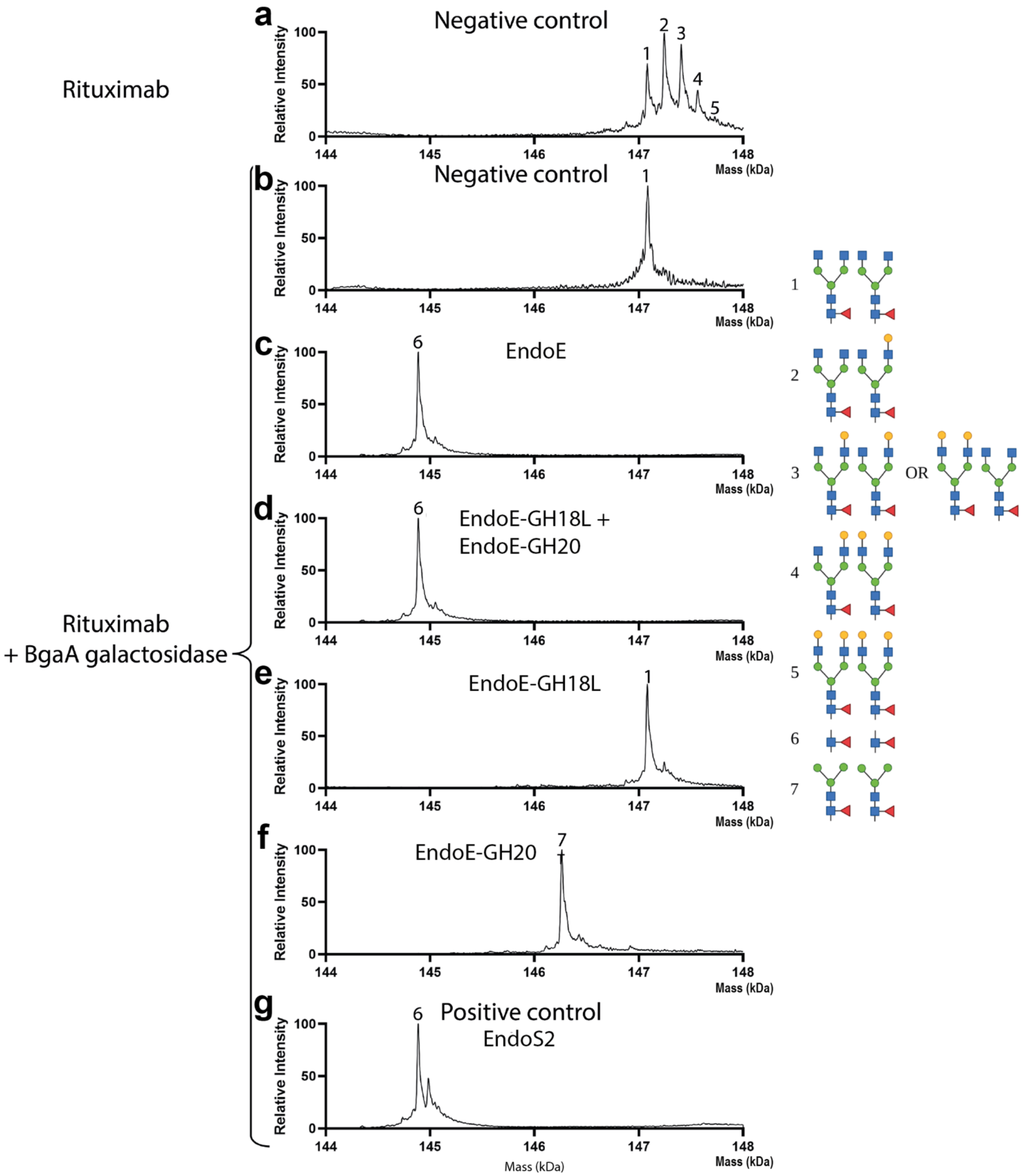

Supplementary Fig. 8 | Molecular basis of EndoE GH18 and GH20 domains substrate specificity as visualized by LC/MS. Rituximab. Processing of CT *N*-glycans on Rituximab + BgaA galactosidase. Mass spectrometry of Rituximab treated with **a** no enzyme (negative control) and Rituximab + BgaA galactosidase treated with **b** no enzyme (negative control) **c** EndoE **d** EndoE-GH18L + EndoE-GH20 **e** EndoE-GH18L **f** EndoE-GH20 and **g** EndoS2 (positive control). The retention time for Rituximab was 2.4 min. For mass deconvolution, the following parameters were used in the BioConfirm software; 2000-7000 m/z and 14.4-14.8 kDa. The theoretical and observed mass of each annotated peak are in Supplementary Table 4.

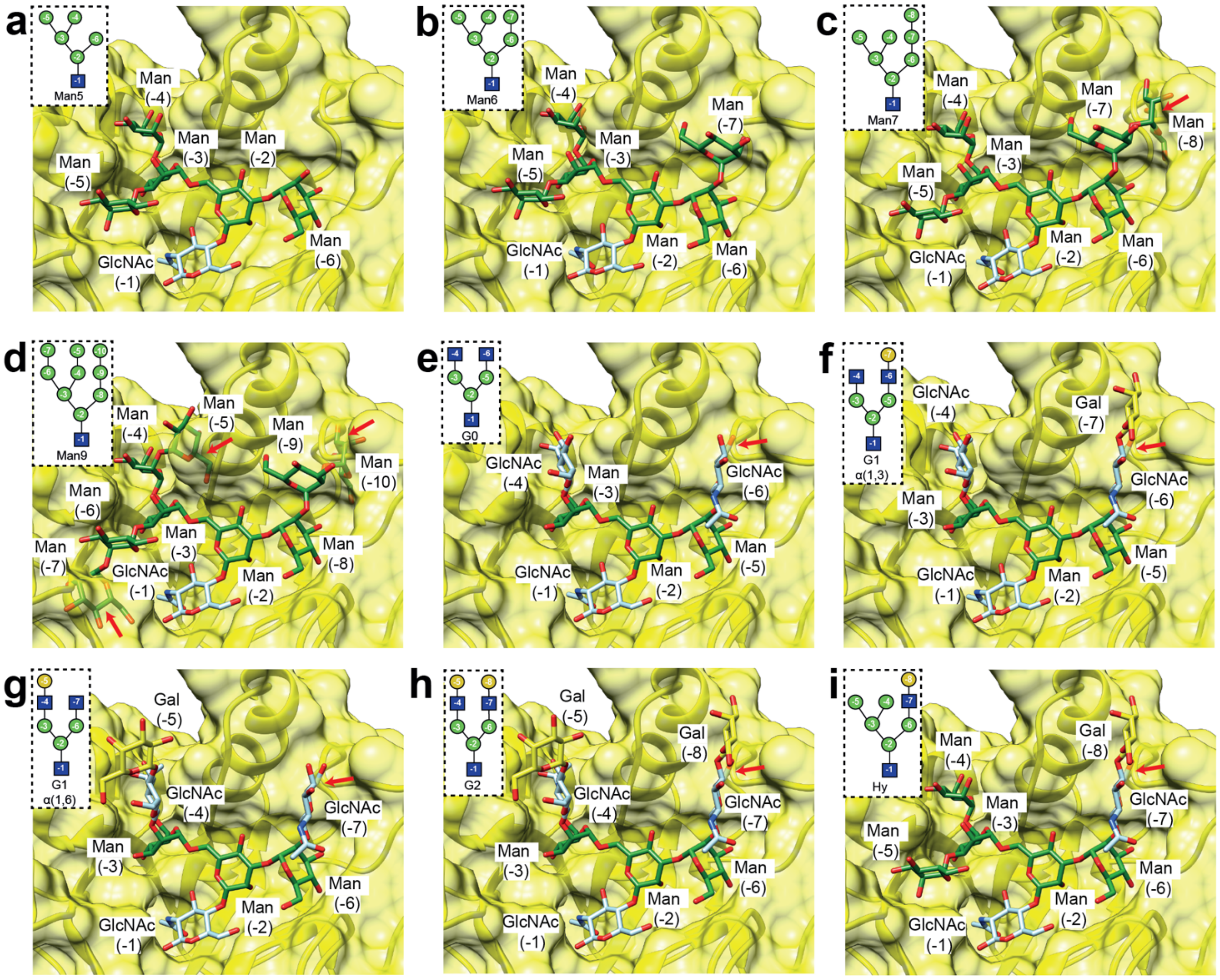

Supplementary Figure 9 | Structural basis of GH18 substrate specificity. a Surface representation of the EndoE-GH18L-Man_5 crystal structure. Molecular docking calculation results of EndoE-GH18L with Man_6 (**b**), Man_7 (**c**), Man_9 (**d**), G0 (**e**), α1-3 G1 (**f**), α1-6 G1 (**g**), G2 (**h**) and Hy (**i**) products. In all the glycan structures, Man(-3) corresponds to the first monosaccharide of the α(1,6) antenna.

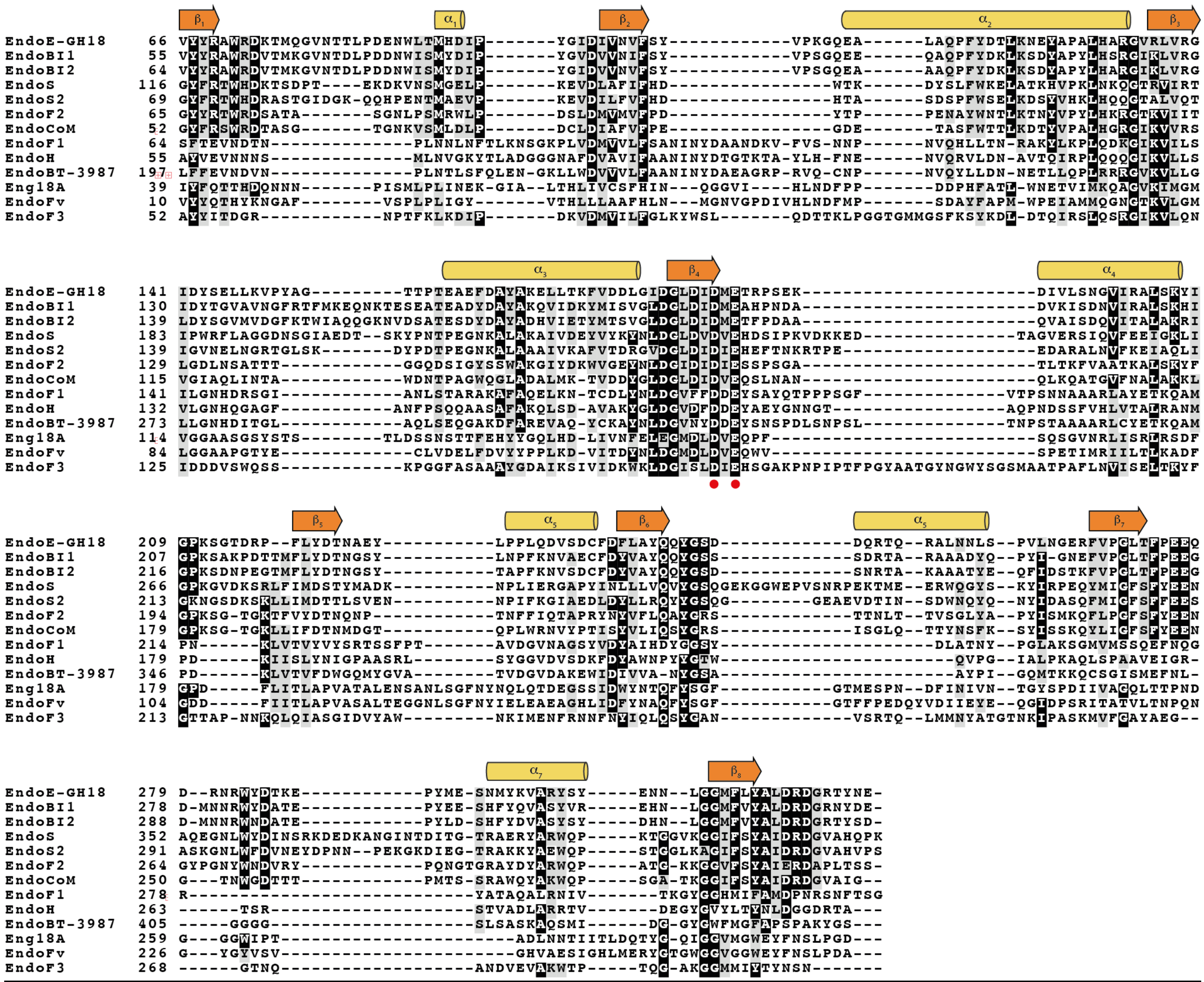

Supplementary Figure 10 | Sequence alignment of EndoE-GH18 with homologues. Comparison of EndoE from *Enterococcus faecalis* (Q6U890, Uniprot code), EndoBI1 and EndoBI2 from *Bifidobacterium longum subsp. infantis* (B7GPC7 and E8MUK6, Uniprot code), EndoS and EndoS2 from *Streptococcus pyogenes serotype M1* (Q99Y92 and T1WGN1, Uniprot code), EndoF1, EndoF2 and EndoF3 from *Elizabethkingia meningoseptica* (P36911, P36912 and P36913, Uniprot code), Endo-CoM from *Cordyceps militaris* (G3JPF7, Uniprot code), EndoH from *Streptomyces plicatus* (P04067, Uniprot code), EndoBT-3987 from *Bacteroides thetaiotaomicron* (Q8A0N4, Uniprot code), Eng18A from *Hypocrea atroviride IMI 206040* (G9NR36, Uniprot code) and EndoFv from *Flammulina velutipes* (D1GA49, Uniprot code). The distribution of secondary structure elements of EndoE-GH18 is displayed above the alignment. Catalytic residues are highlighted with red dots. Amino-acid sequence alignment with other GH18 ENGases revealed that the important residues for substrate binding are conserved in EndoBI1, EndoBI2, EndoS, EndoS2 and EndoF2 and EndoCoM, enzymes that are able to hydrolyze CT-type *N*-glycans.

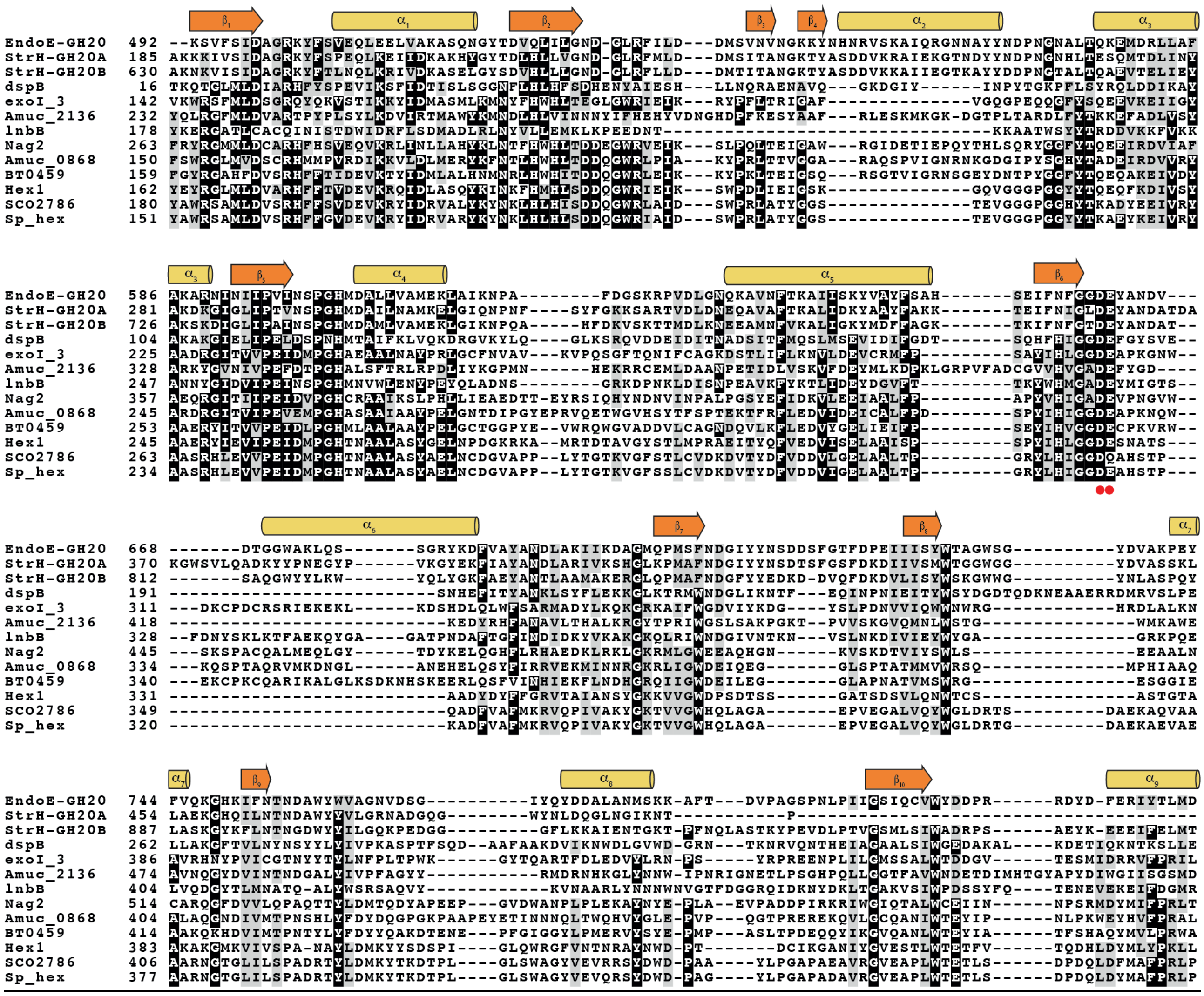

Supplementary Figure 11 | Sequence alignment of EndoE-GH20 with homologues. Comparison of EndoE from *Enterococcus faecalis* (Q6U890, Uniprot code), StrH from *Streptococcus pneumoniae* (P49610, Uniprot code), dispersin B from *Aggregatibacter actinomycetemcomitans* (Q840G9, Uniprot code), β-*N*-acetylhexosaminidase from *Bacteroides fragilis* (D1JST6, Uniprot code), Amuc_2136 from *Akkermansia muciniphila* (B2UPR7, Uniprot code), LNBase from *Bifidobacterium bifidum JCM 1254* (B3TLD6, Uniprot code), Nag2 from *Vibrio harveyi* (D1JST6, Uniprot code), Amuc_0868 from *Akkermansia muciniphila* (7CBO, Uniprot code), BT0459 from *Bacteroides thetaiotaomicron* (A0A174QSL3, Uniprot code), Hex1 from *Paenibacillus sp.* (D0VX21, Uniprot code), SCO2786 from *Streptomyces coelicolor* (Q9L068, Uniprot code) and β-*N*-acetylhexosaminidase from *Streptomyces plicatus* (A0A174QSL3, Uniprot code). The distribution of secondary structure elements of EndoE-GH20 is displayed above the alignment. Catalytic residues are highlighted with red dots.

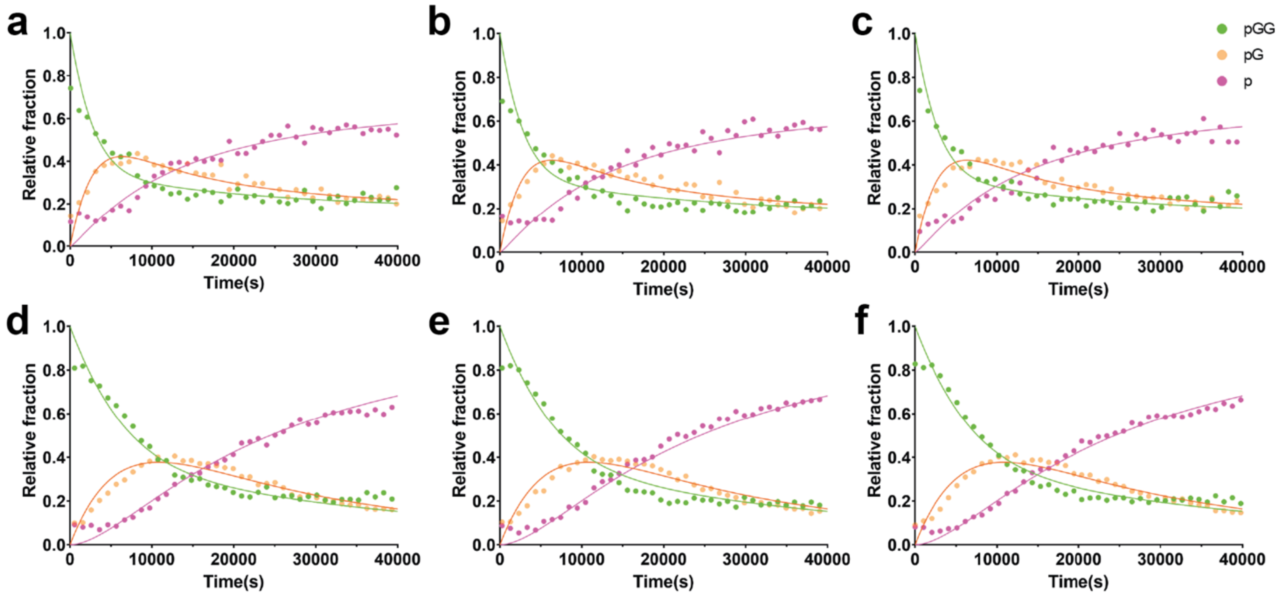

Supplementary Fig. 12 | Kinetic modeling of deglycosylation of G0/G0-Rituximab by EndoE. Kinetic modeling of G0 glycan release from G0/G0-Rituximab by EndoE. Deglycosylation of G0/G0-Rituximab by **a b c** EndoE and **d e f** EndoE-GH18L + EndoE-GH20. Diglycosylated, monoglycosylated and deglycosylated Rituximab is referred to as pGG, pG and p, respectively.
