# Supplementary material for: Mechanism of cooperative N-glycan processing by the multi-modular endoglycosidase EndoE
Source: Nat Commun. 2022 Mar 3;13:1137. doi: 10.1038/s41467-022-28722-w (PMC8894350; doi:10.1038/s41467-022-28722-w)
Supplement: Supplementary file 2 — Reporting Summary [file 41467_2022_28722_MOESM2_ESM.pdf]

nature portfolio

Corresponding author(s): Marcelo E. GUERIN
NCOMMS-21-40384-T

Last updated by author(s): Oct 11, 2021

Reporting Summary

Nature Portfolio wishes to improve the reproducibility of the work that we publish. This form provides structure for consistency and transparency in reporting. For further information on Nature Portfolio policies, see our Editorial Policies and the Editorial Policy Checklist.

Statistics

For all statistical analyses, confirm that the following items are present in the figure legend, table legend, main text, or Methods section.

n/a	Confirmed	
☐	☒	The exact sample size (n) for each experimental group/condition, given as a discrete number and unit of measurement
☐	☒	A statement on whether measurements were taken from distinct samples or whether the same sample was measured repeatedly
☒	☐	The statistical test(s) used AND whether they are one- or two-sided<br>*Only common tests should be described solely by name; describe more complex techniques in the Methods section.*
☒	☐	A description of all covariates tested
☒	☐	A description of any assumptions or corrections, such as tests of normality and adjustment for multiple comparisons
☐	☒	A full description of the statistical parameters including central tendency (e.g. means) or other basic estimates (e.g. regression coefficient) AND variation (e.g. standard deviation) or associated estimates of uncertainty (e.g. confidence intervals)
☒	☐	For null hypothesis testing, the test statistic (e.g. F, t, r) with confidence intervals, effect sizes, degrees of freedom and P value noted<br>*Give P values as exact values whenever suitable.*
☒	☐	For Bayesian analysis, information on the choice of priors and Markov chain Monte Carlo settings
☒	☐	For hierarchical and complex designs, identification of the appropriate level for tests and full reporting of outcomes
☒	☐	Estimates of effect sizes (e.g. Cohen's d, Pearson's r), indicating how they were calculated

Our web collection on statistics for biologists contains articles on many of the points above.

Software and code

Policy information about availability of computer code

Data collection	Agilent 1290 Infinity II LC System equipped with a 50 mm PLRP-S column from Agilent with 1000 Å pore size. The LC system is attached to an Agilent 6560 Ion Mobility (IM) quadrupole- time of flight (Q-TOF) mass spectrometer (Agilent, Santa Clara, CA)
Data analysis	xds VERSION Feb 5, 2021<br>ccp4-7.0.078<br>phenix-1.18.2-3874<br>coot 0.8.9.2<br>chimera 1.14<br>glycam-web (https://glycam.org)<br>autodock vina 1.1.2

For manuscripts utilizing custom algorithms or software that are central to the research but not yet described in published literature, software must be made available to editors and reviewers. We strongly encourage code deposition in a community repository (e.g. GitHub). See the Nature Portfolio guidelines for submitting code & software for further information.

Data

Policy information about availability of data

All manuscripts must include a data availability statement. This statement should provide the following information, where applicable:

- Accession codes, unique identifiers, or web links for publicly available datasets
- A description of any restrictions on data availability
- For clinical datasets or third party data, please ensure that the statement adheres to our policy

The atomic coordinates and structure factors have been deposited with the Protein Data Bank, accession codes 7PUJ [http://dx.doi.org/10.2210/pdb7PUJ/pdb] (EndoE-GH18L), 7PUK [http://dx.doi.org/10.2210/pdb7PUK/pdb] (EndoE-GH18L-Man5), and 7PUL [http://dx.doi.org/10.2210/pdb7PUL/pdb] (EndoE-GH20). Data are available from the corresponding authors upon reasonable request.

Field-specific reporting

Please select the one below that is the best fit for your research. If you are not sure, read the appropriate sections before making your selection.

[x] Life sciences [ ] Behavioural & social sciences [ ] Ecological, evolutionary & environmental sciences

For a reference copy of the document with all sections, see nature.com/documents/nr-reporting-summary-flat.pdf

Life sciences study design

All studies must disclose on these points even when the disclosure is negative.

Sample size	No sample size calculation was performed.
Data exclusions	No data was excluded from the activity experiments.
Replication	All LC-MS enzymatic activity assays were performed in triplicate. All attempts at replication were successful.
Randomization	This is not relevant for this study
Blinding	Blinding is not relevant to your study

Reporting for specific materials, systems and methods

We require information from authors about some types of materials, experimental systems and methods used in many studies. Here, indicate whether each material, system or method listed is relevant to your study. If you are not sure if a list item applies to your research, read the appropriate section before selecting a response.

Materials & experimental systems

n/a	Involved in the study	
[ ]	[x]	Antibodies
[x]	[ ]	Eukaryotic cell lines
[x]	[ ]	Palaeontology and archaeology
[x]	[ ]	Animals and other organisms
[x]	[ ]	Human research participants
[x]	[ ]	Clinical data
[x]	[ ]	Dual use research of concern

Methods

n/a	Involved in the study	
[x]	[ ]	ChIP-seq
[x]	[ ]	Flow cytometry
[x]	[ ]	MRI-based neuroimaging

Antibodies

Antibodies used	Rituximab (RITUXAN, Genentech) was kindly provided courtesy of the University of Maryland Greenebaum Comprehensive Cancer Center.
Validation	*Describe the validation of each primary antibody for the species and application, noting any validation statements on the manufacturer's website, relevant citations, antibody profiles in online databases, or data provided in the manuscript.*
